# Gut microbiota-mediated C-sulfonate metabolism impairs the bioavailability and anti-cholestatic efficacy of andrographolide

**DOI:** 10.1080/19490976.2024.2387402

**Published:** 2024-09-12

**Authors:** Dafu Tang, Wanyu Hu, Bingxuan Fu, Xiaojie Zhao, Guoquan You, Cong Xie, Hong Yu Wang, Xueni Guo, Qianbing Zhang, Zhongqiu Liu, Ling Ye

**Affiliations:** a NMPA Key Laboratory for Research and Evaluation of Drug Metabolism & Guangdong Provincial Key Laboratory of New Drug Screening, School of Pharmaceutical Sciences, Southern Medical University, Guangzhou, China; b Clinical Pharmacy Center, Nanfang Hospital, Southern Medical University, Guangzhou, China; c Cancer Research Institute, School of Basic Medical Sciences, Southern Medical University, Guangzhou, China; d International Institute for Translational Chinese Medicine, Guangzhou University of Chinese Medicine, Guangzhou, Guangdong, China

**Keywords:** Andrographolide, C-sulfonate metabolism, gut microbiota, oral bioavailability, anti-cholestatic effect, APS reductase, *Desulfovibrio piger*, FXR

## Abstract

Cholestatic liver injury results from the accumulation of toxic bile acids in the liver, presenting a therapeutic challenge with no effective treatment available to date. Andrographolide (AP) has exhibited potential as a treatment for cholestatic liver disease. However, its limited oral bioavailability poses a significant obstacle to harnessing its potent therapeutic properties and restricts its clinical utility. This limitation is potentially attributed to the involvement of gut microbiota in AP metabolism. In our study, employing pseudo-germ-free, germ-free and strain colonization animal models, along with 16S rRNA and shotgun metagenomic sequencing analysis, we elucidate the pivotal role played by gut microbiota in the C-sulfonate metabolism of AP, a process profoundly affecting its bioavailability and anti-cholestatic efficacy. Subsequent investigations pinpoint a specific enzyme, adenosine-5’-phosphosulfate (APS) reductase, predominantly produced by *Desulfovibrio piger*, which catalyzes the reduction of SO_4_^2-^ to HSO_3_^−^. HSO_3_^−^ subsequently interacts with AP, targeting its C=C unsaturated double bond, resulting in the formation of the C-sulfonate metabolite, 14-deoxy-12(R)-sulfo andrographolide (APM). Inhibition of APS reductase leads to a notable enhancement in AP bioavailability and anti-cholestatic efficacy. Furthermore, employing RNA sequencing analysis and farnesoid X receptor (FXR) knockout mice, our findings suggest that AP may exert its anti-cholestatic effects by activating the FXR pathway to promote bile acid efflux. In summary, our study unveils the significant involvement of gut microbiota in the C-sulfonate metabolism of AP and highlights the potential benefits of inhibiting APS reductase to enhance its therapeutic effects. These discoveries provide valuable insights into enhancing the clinical applicability of AP as a promising treatment for cholestatic liver injury.

## GRAPHICAL ABSTRACT

**Figure:**
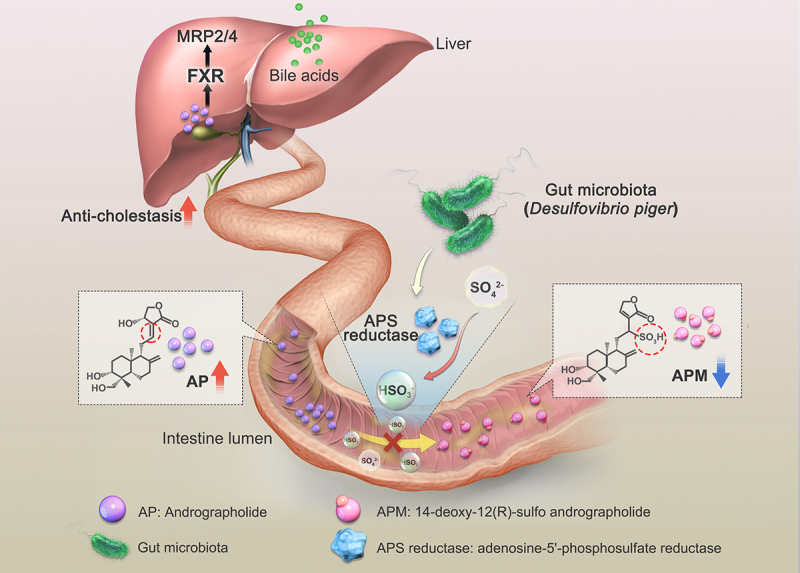

## 1. Introduction

Cholestasis represents a pivotal characteristic of cholestatic liver disease, and its untreated progression can lead to severe liver damage and end-stage liver diseases, thus constituting a significant public health concern.^1,2^ The report indicates that cholestasis occurs in approximately 35% of patients with chronic liver disease, with a higher prevalence in individuals diagnosed with primary biliary cholangitis and primary sclerosing cholangitis.^3^ Currently, therapeutic options for cholestatic liver disease are limited, with only ursodeoxycholic acid (UDCA) and obeticholic acid (OCA) approved by the Food and Drug Administration (FDA).^4^ Nevertheless, not all patients respond favorably to these treatments. UDCA is the first-line therapy in primary biliary cholangitis, the side-effects of UDCA treatment in most patients include bloating, weight gain, and sometimes self-reported thinning of hair.^5,6^ While OCA is currently approved for second-line therapy in patients with primary biliary cholangitis who have incomplete responses to UDCA or who are intolerant of UDCA, treatment with OCA is associated with a dose-dependent increase in pruritus, reported in roughly 40% of treated patients, which is the leading cause for drug discontinuation.^7,8^ S-adenosyl methionine (S-AMe) serves as a second-line treatment for cholestatic disease, primarily effective in the earlier stages of the disease.^9^ Hence, the development of novel therapeutic agents for cholestatic liver disease is of paramount importance. Natural products have demonstrated their potential as valuable sources of anti-cholestasis agents, offering a potential shortcut in the quest for effective treatments.^10^

Andrographolide (AP), the primary active compound found in *Andrographis paniculata* (Burm. f.) Nees, has been documented to exhibit protective and therapeutic effects against liver diseases,^11^ attributed to its well-known anti-inflammatory,^12^ anti-oxidative,^13^ and anti-fibrosis properties.^14^ Recent studies have revealed significant protective effects of AP against α-naphthylisothiocyanate (ANIT)-induced cholestatic liver injury in rats.^15,16^ Additionally, AP serves as a quality control component for Xiaoyan Lidan Formula,^17^ a classic Traditional Chinese Medicine formula known for its efficacy in alleviating various hepatobiliary disorders, including cholestasis, jaundice, and hepatitis in clinical practice.^18^ Except for *Andrographis paniculata* (Burm. f.) Nees (known as Chuanxinlian), this formula comprises two other Chinese herbs: Herba Rabdosia (the dried herb of *Rabdosia serra* (Maxim.) Hara, known as Xihuangcao), and Ramulus et Folium Picrasmae (the dried twigs and leaves of *Picrasma quassioides* (D. Don) Benn, known as Kumu).^19^ However, the clinical application of AP is severely hampered by its extremely low bioavailability, with rat absolute oral bioavailability ranging from 1.19% to 2.67%.^20–22^ Our previous study has illuminated that AP undergoes C-sulfonate metabolism in the rat intestine, resulting in the formation of a significant quantity of C-sulfonate metabolite 14-deoxy-12(R)-sulfo andrographolide (APM), which predominantly accounts for its poor oral bioavailability.^22^ Currently, the formulation of AP into solid lipid nanoparticles, a dosage form modification, offers a partial improvement in bioavailability, but AP still undergoes substantial metabolism into APM within the intestinal tract.^23^ Consequently, addressing the inhibition of metabolism and enhancement of AP’s bioavailability has emerged as an urgent challenge.

C-sulfonate metabolism denotes a process wherein sulfur atoms with lone pairs attack the β-carbon atoms of α, β-unsaturated carbonyl compounds to yield sulfonate compounds, although its precise mechanism remains elusive.^22,24^ This type of metabolism extends beyond AP and has been observed in various other compounds, including physalin A,^25^ nitecapone,^26^ 2-pyridinethiol-1-oxide,^27^ and flumipropyn.^28^ Sulfonate metabolism can be mediated by host-derived sulfotransferases (SULTs) for O- and N-sulfonates,^29^ but likely not for C-sulfonates.^30^ It is highly probable that the gut microbiota contributes sulfite or bisulfite to mediate C-sulfonate metabolism, resulting in the generation of C-sulfonate metabolites.^31^

The gut microbiota serves as a pivotal “metabolic organ”, playing a crucial role in drug metabolism by harboring a diverse range of enzymes and metabolic pathways capable of both activating and inactivating drugs, which has significant implications for drug efficacy and safety.^32,33^ For instance, certain drugs undergo conversion into their active forms through gut microbiota-mediated metabolism, enhancing their pharmacological effects. For example, the gut microbiota facilitates the conversion of the prodrug sulfasalazine into its active anti-inflammatory component, 5-aminosalicylate,^34^ and sulindac into its active form, sulindac sulfide.^35^ Conversely, the gut microbiota can also metabolize drugs into inactive forms, thereby reducing their pharmacological activity. Specific gut bacteria, such as *Lysinibacillus sphaericus*, possess the ability to metabolize aspirin, decreasing its bioavailability.^36^ Additionally, microbiome-derived enzymes, such as gut commensal acetyltransferases, inactivate 5-aminosalicylic acid, consequently reducing its therapeutic efficacy.^37^ Moreover, the gut microbiota can diminish the effectiveness of the antidiabetic drug acarbose through microbial enzymes.^38^ This intricate interplay underscores the importance of considering microbiota-mediated metabolism in understanding drug responses and optimizing therapeutic outcomes. Consequently, further investigation is imperative to determine whether the gut microbiota is involved in the C-sulfonate metabolism of AP and its potential impact on AP’s bioavailability and efficacy.

The primary objective of our study was to examine the impact of gut microbiota-mediated C-sulfonate metabolism of AP on both its bioavailability and anti-cholestatic efficacy. Employing pseudo-germ-free, germ-free and strain colonization animal models, along with 16S rRNA and shotgun metagenomic sequencing analysis, we uncovered a significant revelation that the gut microbiota plays a pivotal role in the C-sulfonate metabolism of AP, subsequently influencing its bioavailability and anti-cholestatic effect. Furthermore, we identified adenosine-5’-phosphosulfate (APS) reductase as an enzyme primarily secreted by *Desulfovibrio* spp., particularly *Desulfovibrio piger* (*D. piger*), providing HSO_3_^−^, actively participating in the C-sulfonate metabolism of AP. Inhibiting APS reductase resulted in enhanced bioavailability and anti-cholestatic effect of AP. Moreover, utilizing RNA sequencing analysis and farnesoid X receptor (FXR) knockout mice, we delved into elucidating the potential mechanisms underlying AP’s anti-cholestatic effect through the activation of FXR pathway to promote bile acid efflux. In summary, our study elucidates the pivotal role of gut microbiota in the C-sulfonate metabolism of AP, emphasizing the potential advantages of APS reductase inhibitor to enhance its therapeutic effectiveness.

## 2. Materials and methods

### 2.1. Chemicals and reagents

Andrographolide (AP, purity > 98%) and genistein (purity >98%, as an internal standard (IS)) were obtained from Chengdu Pufeide Co., Ltd (Chengdu, China). 14-deoxy-12(R)-sulfo andrographolide (APM) was synthesized and identified by our laboratory according to a previously described procedure^24,39^ and its purity was above 98%. APS reductase inhibitor bromocriptine mesylate (Inhibitor) was obtained from Shanghai Yuanye Biotechnology Co., Ltd (Shanghai, China). S-adenosyl methionine (S-AMe, as a positive drug), α-naphthylisothiocyanate (ANIT), sodium sulfate (Na_2_SO_4_), and sodium bisulfite (NaHSO_3_) were from Sigma-Aldrich Company (St. Louis, MO, USA). Ampicillin, vancomycin, neomycin sulfate, metronidazole, ethylenediaminetetraacetic acid disodium salt (EDTA-Na2), basic fuchsin were from Dalian Meilun Biotechnology Co., Ltd (Dalian, China). Pooled rat liver S9 fraction (RLS9) and pooled mouse liver S9 fraction (MLS9) were prepared based on the method used in previous study in our lab.^40^ Pooled human liver S9 fraction (HLS9) was from BD Biosciences (Woburn, MA). Other reagents were of analytical grade or better and were commercially available.

### 2.2. Animals

C57BL/6J male mice (18–22 g, 6–8 weeks old) and Sprague-Dawley (SD) male rats (180–220 g, 6–8 weeks old) were obtained from the Experimental Animal Center of Southern Medical University (Guangzhou, China) (License number: SCXK(Yue)-2016–0041). Germ-free C57BL/6J male mice (18–22 g, 6–8 weeks old) were obtained from the Experimental Animal Center of the First Affiliated Hospital of Sun Yat-sen University (Guangzhou, China) (License number: SYXK(Yue)-2020–0233). The wild-type (WT) mice and the FXR knockout (*Fxr*^*-/-*^) mice (18–22 g, 6–8 weeks old) were both on a C57BL/6J genetic background.^41^ All animals were housed in a specific pathogen-free (SPF) room with a temperature of 25–26°C and a 12-h light/dark cycle. Germ-free mice were raised in special sterile isolators. All animals underwent a one-week acclimation period to adapt to these conditions before commencing the experiments. During the experimental period, all animals were provided unrestricted access to both food and autoclaved water. This study was conducted in strict adherence to the “Guide for the Care and Use of Laboratory Animals” and received approval from the Institutional Animal Ethics Committee of Southern Medical University (Guangzhou, China).

### 2.3. Animals treatments

#### 2.3.1. Antibiotics (ABX) treatment

C57BL/6J male mice and SD male rats were treated with antibiotics (ABX) to deplete gut microbiota. All animals were given autoclaved water supplemented with ampicillin (1 g/L) and received a daily oral gavage of an antibiotic mixture consisting of vancomycin (50 mg/kg), neomycin sulfate (100 mg/kg), and metronidazole (100 mg/kg) for a duration of 2 weeks. The establishment of the ABX (pseudo-germ-free) model has been successfully identified by the cultivation of viable bacteria (Supplementary Figure S1) and 16S rRNA sequencing.^42^

#### 2.3.2. *Desulfovibrio piger* (*D.* *piger*) culture and treatment

*D. piger* (DSM 749) obtained from Ningbo MingZhou Bio Co.,Ltd. (Zhejiang, China) was cultured at 37°C in DSMZ Medium 63 (ShanDong Tuopu Bio-engineering Co., Ltd.) following the supplier’s instructions. The concentration of *D. piger* was measured at OD600 using a Tecan Infinite M1000 PRO Multifunctional Enzyme Labeler (Tecan, Switzerland). For *D. piger* species colonization experiment, bacterial cells were harvested by centrifugation and resuspended in PBS. Mice were orally administered 0.2 mL of the *D. piger* suspension (1 × 10^9^ CFU/mL) daily for 2 weeks. The colonization of *D. piger* in mice was evaluated using real-time quantitative PCR.^43^

#### 2.3.3. Pharmacodynamic experiments

To investigate the impact of gut microbiota on the anti-cholestatic effect of AP, Control and ABX mice were divided into five groups, respectively: Vehicle, AP (200 mg/kg), ANIT (50 mg/kg), ANIT+AP (200 mg/kg), and ANIT+S-AMe (100 mg/kg).

Similarly, to investigate the impact of C-sulfonate metabolism on the anti-cholestatic effect of AP, mice were randomly divided into two groups: p.o. group (oral administration) and i.p. group (intraperitoneal injection). The p.o. group was further divided into six subgroups: Vehicle, AP (400 mg/kg), ANIT (50 mg/kg), ANIT+AP (100 mg/kg), ANIT+AP (200 mg/kg), ANIT+AP (400 mg/kg). While the i.p. group was divided into five subgroups: Vehicle, AP (200 mg/kg), ANIT (50 mg/kg), ANIT+AP (100 mg/kg), ANIT+AP (200 mg/kg).

Additionally, to investigate the effect of APS reductase inhibitor on the anti-cholestatic effect of AP, mice were randomly divided into Control and Inhibitor groups, each further divided into five subgroups: Vehicle, AP (200 mg/kg), ANIT (50 mg/kg), ANIT+AP (200 mg/kg), and ANIT+S-AMe (100 mg/kg). The mice received APS reductase inhibitor bromocriptine mesylate by oral gavage (20 mg/kg, p.o.) 2 weeks before AP treatment and continued until the end of the experiment. Furthermore, to investigate the important role of FXR in the anti-cholestatic effect of AP, WT and *Fxr*^*-/-*^ mice were, respectively, divided into the following groups: Vehicle, ANIT (50 mg/kg), and ANIT+AP (200 mg/kg).

In pharmacodynamic experiments, mice were administered AP at various dosages through different routes daily for a duration of 2 weeks. Additionally, S-AMe was administered at a dosage of 100 mg/kg via intraperitoneal injection (i.p.) daily for 2 weeks. A single dose of ANIT (50 mg/kg, p.o.) was administered 48 h before euthanizing the mice. At the conclusion of each experiment, the mice were euthanized, and blood samples were collected for biochemical analysis and tissue samples for histological examination and UPLC-MS/MS analysis.

#### 2.3.4. Pharmacokinetics experiments

Control and ABX rats were orally administered a single dose of AP at 120 mg/kg. Blood samples (500 μL) were collected at predetermined time intervals (0, 5, 10, 20, 30, 40, 60, 90, 120, 180, 240, 360, and 480 min). In some cases, APS reductase inhibitor bromocriptine mesylate (20 mg/kg, p.o.) or Na_2_SO_4_ (200 mg/kg, p.o.) was given to rats 1 week before AP treatment.

Mice were orally administered APS reductase inhibitor bromocriptine mesylate (20 mg/kg, p.o.) or Na_2_SO_4_ (200 mg/kg, p.o.) or saline solution 1 week prior to AP treatment (a single dose of AP at 100 mg/kg, p.o.). Blood samples (50 μL) were collected at predetermined time points (0, 10, 20, 40, 60, 90, 120, 240, 360 min). In specific cases, mice were orally administrated with *D. piger* suspension (0.2 mL, 1 × 10^9^ CFU/mL) or saline solution by oral gavage for 2 weeks before AP treatment (a single dose of AP at 100 mg/kg, p.o.).

The pharmacokinetic parameters of AP including peak time (*T*_max_), maximum concentration (*C*_max_), area under the curve from zero time to the last time point measured (*AUC*_0-t_), area under the curve from zero time to infinity (*AUC*_0-∞_), and elimination half-life (*t*_1/2_) were estimated using DAS 2.0 software (Shanghai, China). The relative oral bioavailability (*F*_r_) of AP was calculated by the following equation:

$$F_{r}\;(\%)=\frac{\mathrm{AU}C_{0\;-\infty }{of\;ABX/I\mathrm{nhibitor}/SO}_{4}^{2\;-}/D.\mathrm{piger}}{\mathrm{AU}C_{0\;-\infty }\mathrm{of}\;\mathrm{Control}}\times \;100\%$$

#### 2.3.5. Tissue distribution experiments

Control and ABX rats were orally administered a single dose of AP at 120 mg/kg. Control, ABX and germ-free C57BL/6J mice were orally administered a single dose of AP at 100 mg/kg. After 90 min of AP administration, all animals were euthanized, and blood and tissue samples were collected for subsequent analysis.

Mice were orally administered APS reductase inhibitor bromocriptine mesylate (20 mg/kg, p.o.) or Na_2_SO_4_ (200 mg/kg, p.o.) or saline solution 1 week before AP treatment (a single dose of AP at 100 mg/kg, p.o.). After 360 min of AP treatment, all mice were euthanized, and tissue samples were collected for subsequent analysis. In specific cases, mice were orally administered *D. piger* suspension (0.2 mL, 1 × 10^9^ CFU/mL) or saline solution 2 weeks before AP treatment (a single dose of AP at 100 mg/kg, p.o.).

### 2.4. Co-culturing AP with cecum samples or *D.* *piger*

The cecum contents from mice were collected and mixed with sterilized anaerobic medium in a ratio of 1.0 g to 10 mL, with gentle agitation. After filtered, the mixed medium containing the intestinal flora was placed in an anaerobic environment and preincubated at 37°C for 30 min before use. Mixed medium with or without APS reductase inhibitor bromocriptine mesylate (20 μg/mL) was incubated with AP (10 μg/mL), while autoclaved mixed medium without APS reductase inhibitor served as the control. Incubation was performed in a shaking incubator at 37°C for 6 h, with strict maintenance of anaerobic condition throughout the process.^44^ Similarly, *D. piger* (0.2 mL, 1 × 10^8^ CFU/mL) with or without inhibitor, was incubated with AP for 0, 3, 6, and 12 h, with autoclaved *D. piger* without inhibitor serving as the control. The reaction was terminated by adding cold methanol contained IS. And then samples were analyzed by UPLC-MS/MS to detect the concentrations of AP and APM. Concentrations of APS reductase and HSO_3_^−^ were also determined at the end of incubation.

### 2.5. Biochemical analysis and histological examination

Biochemical indicators of cholestatic liver injury, including alanine aminotransferase (ALT), aspartate aminotransferase (AST), total bile acid (TBA), and total bilirubin (TBIL), were assessed using assay kits obtained from Nanjing JianCheng Bioengineering Institute (Nanjing, China). The experimental procedures were conducted in accordance with the protocols provided by the manufacturer. Fresh liver tissues were fixed in 10% formalin, and prepared as 4-μm paraffin sections. These sections were stained with hematoxylin and eosin for examination of liver histopathology. The stained tissue sections were then observed and captured under an optical microscope for further analysis. Liver histological changes were quantified using a slightly modified scoring system published previously.^45^ The scoring system comprised four histological features that were evaluated semi-quantitatively: necrosis and fibrosis (0–4), steatosis (0–3), lobular inflammation (0–2), and hepatocellular ballooning (0–2). The liver necrosis and fibrosis areas were quantified by area fraction analysis (Image J software, V1.8.0, National Institutes of Health, USA).^46^

### 2.6. 16S rRNA sequencing

The 16S rRNA sequencing of fecal samples obtained from Control and ABX-treated mice was conducted with slight modifications following our previously described protocol.^43^ Total microbial genomic DNA was extracted from fecal samples using the PF Mag-Bind Stool DNA Kit (Omega Bio-tek, Georgia, USA). DNA quality and concentration were determined by 1.0% agarose gel electrophoresis using a NanoDrop® ND-2000 spectrophotometer (Thermo Scientific Inc., USA). The hypervariable region V3-V4 of the bacterial 16S rRNA gene was amplified using primer pairs 338F (5’-ACTCCTACGGGAGGCAGCAG-3’) and 806 R (5’-GGACTACHVGGGTWTCTAAT-3’) by the ABI GeneAmp® 9700 PCR thermocycler (ABI, CA, USA). Purified amplicons were pooled in equimolar amounts and paired-end sequenced on an Illumina PE300/PE250 platform (Illumina, San Diego, USA) according to the standard protocols by Majorbio Bio-Pharm Technology Co. Ltd. (Shanghai, China). Raw fastq files were de-multiplexed and quality-filtered using QIIME, and the sequences were matched against a high-quality 16S rRNA sequence from the Green Genes database after trimming the primer, barcode, and chimeras. Then, the optimized sequences were clustered into operational taxonomic units (OTUs) using UPARSE 11 with 97% sequence similarity level. Finally, we performed the data analysis.

### 2.7. Shotgun metagenomic sequencing

Shotgun metagenomic sequencing was performed using cecum samples from mice after AP treatment (400 mg/kg, oral, 14 d). DNA was extracted by a PF Mag-Bind DNA Kit (Omega Bio-tek, USA) and qualified by 1% Agarose gel electrophoresis. Extracted DNA was fragmented by Covaris M220 (Gene Company Limited, China) and screened at approximately 400 bp fragments for paired-end library construction. NEXTFLEX® Rapid DNA-Seq (Bioo Scientific, USA) was used to build paired-end library construction. The Illumina NovaSeq 6000 (Illumina, USA) sequencing platform was used for metagenomic sequencing. Fastp was used to perform quality cropping on the adapter sequences of the 3’ and 5’ ends of reads, preserving high-quality paired-end reads and single-end reads. BWA (version 0.7.17) was used to construct the reads with the host DNA sequence and remove contaminated reads with high alignment similarity. MEGAHIT (version 1.1.2) was used to splice and assemble the optimized sequence. SOAP aligner (version soap2.21release) was used to compare the high-quality reads of each sample with the non-redundant gene set (95% identity) and calculate the abundance information of genes in the corresponding samples. DIAMOND (version 2.0.11) was used to annotate species taxonomy, and KEGG function. Data was deposited into the NCBI Sequence Read Archive (SRA) database.

### 2.8. RNA sequencing analysis

RNA in liver tissue was isolated from mice treated with ANIT and ANIT+AP (200 mg/kg, i.p.) and purified by TRIzol (Invitrogen, CA). The isolated RNA was quantified using Qubit 2.0 (Thermo Fisher Scientific, USA) following the manufacturer’s protocol. Libraries were prepared using the NEBNext® UltraTM RNA Library Prep Kit for Illumina® (NEB, USA) and sequenced on a Novaseq 6000 (Illumina, USA) at Novogene’s deep sequencing facility. Mapped reads from each sample were assembled using StringTie (v1.3.3b) in a reference-based approach, and featureCounts was used to quantify mapped reads. Differential expression analysis of two conditions/groups was performed using the DESeq2 R package (1.20.0).

### 2.9. UPLC-MS/MS analysis

An ACQUITY^TM^ UPLC-MS/MS system equipped with an ACQUITY^TM^ UPLC BEH C_18_ Column (50 × 2.1 mm I.D., 1.7 μm, Waters, Milford, MA, USA) was utilized for the quantification of AP and APM as previously described.^47^ Briefly, the detection samples (50 μL) were mixed with 50 μL of IS (50 ng/mL). AP and APM were extracted by 400 μL cold methanol. Supernatants were dried under nitrogen stream. Then, the residue was dissolved with 50% methanol in water, centrifuged for 30 min at 13,000 rpm, and 10 µL of the supernatant was subjected to UPLC-MS/MS analysis. The mobile phases consisted of eluent A (water) and eluent B (methanol) with a gradient elution as follows: 0–0.8 min, 10% B; 0.8–1.2 min, 10–90% B; 1.2–5.0 min, 90% B; 5.0–5.5 min, 90–10% B; 5.5–7.0 min, 10% B. The flow rate was 0.3 mL/min and the injection volume of all samples was 10 μL. The quantification of AP, APM and IS was performed by UPLC-MS/MS using an MRM method with the transitions of *m/z* 349.1→*m/z* 287.2 for AP, *m/z* 413.2→*m/z* 287.2 for APM, *m/z* 269→*m/z* 133 for IS.

### 2.10. APS reductase and HSO_3_^−^ concentration determination

For the determination of APS reductase concentration, the supernatant from mouse cecum contents and the *D. piger* strain were assessed using microorganism APS reductase elisa assay kit (Shanghai Senxingyan Biotechnology Co., Ltd., China), following the instructions provided with the kit. For the determination of HSO_3_^−^ concentration, cecum contents from mice or rats were weighed and homogenized in five volumes of normal saline (based on tissue weight). The homogenate was then subjected to centrifugation at 13,000 rpm and 4°C for 15 min. The supernatant (or perfusate) was collected and mixed with 0.2% formaldehyde, 0.05% basic fuchsin, and 0.01% EDTA-Na2 in a ratio of 1:1:1:9 under acidic conditions. To establish a standard curve, different concentrations of NaHSO_3_ were used. Subsequently, the absorbance of the resulting solutions was measured at a wavelength of 560 nm after 40 min.

### 2.11. Real-time quantitative PCR (qPCR) analysis

Total RNA from frozen livers of Vehicle, AP (200 mg/kg, i.p.), ANIT (50 mg/kg), and ANIT+AP (200 mg/kg, i.p.) groups was extracted using the Animal Total RNA Isolation Kit (FOREGENE, China). cDNA was synthesized using a Color Reverse Transcription Kit (EZBioscience, Roseville, CA, USA). qPCR was performed using SYBR green/Rox qPCR master mix (EZBioscience, Roseville, CA, USA), according to the manufacturer’s instruction. The expression of target genes was normalized to *β-actin*, and fold changes were calculated using 2^–ΔΔCT^ method. For bacterial identification, genomic DNA of fecal flora was isolated using the TIANamp Stool DNA Kit (TIANGEN, Beijing, China) and qPCR was performed. The relative abundance and expression of *Desulfovibrio* spp., *D. piger* and APS reductase were normalized to the amounts of total bacteria (16S). All primers were synthesized by Tsingke Biotechnology Co., Ltd. (Beijing, China) and were listed in Supplementary Table S1.

### 2.12. Western blot analysis

Liver tissue from Vehicle, AP (200 mg/kg, i.p.), ANIT (50 mg/kg), and ANIT+AP (200 mg/kg, i.p.) groups was homogenized in RIPA buffer (Thermo Scientific). Homogenized liver tissue was then incubated on ice for 1 h and then centrifuged at −4°C for 20 min at 13,000 rpm. The collected supernatant was quantified using BCA protein assay kit (Epizyme). Primary antibodies including anti-FXR (anti-NR1H4, bs-12,867 R, bioss), anti-MRP2 (anti-ABCC2, bs-1092 R, bioss), anti-MRP3 (anti-ABCC3, 39909S, CST), anti-MRP4 (anti-ABCC4, DF6921, Affinity), anti-OATP1 (anti-SLCO1C1, bs -11,436 R, bioss), NTCP Polyclonal Antibody (ABP53103, Abbkine) and GADPH (60004–1-Ig, proteintech) were incubated overnight at 4°C. The sections were incubated for 1 h at room temperature with a secondary antibody diluted 1:5000. The membrane was then washed three times with TBST for 5 min. To visualize the protein bands, the membrane was reacted with Immobilon Forte Western HRP (Millipore) substrate solution for 2 min and analyzed using Tanon5200 imager. Data analysis was performed using ImageJ software (V1.8.0, National Institutes of Health, USA).

### 2.13. Statistical analysis

Data analysis and graphing were conducted by GraphPad Prism v.8 (GraphPad, USA). Two-tailed unpaired Student’s *t*-test was used to evaluate the significant difference between two groups, while one-way ANOVA followed by Tukey’s multiple comparisons test was applied for three or more groups. Results were presented as mean ± standard error of the mean (SEM) or mean ± standard deviation (SD) and *p* ＜ 0.05 was considered statistically significant.

## 3. Results

### 3.1. Gut microbiota depletion enhances the anti-cholestatic effect of AP

**Figure 1.**
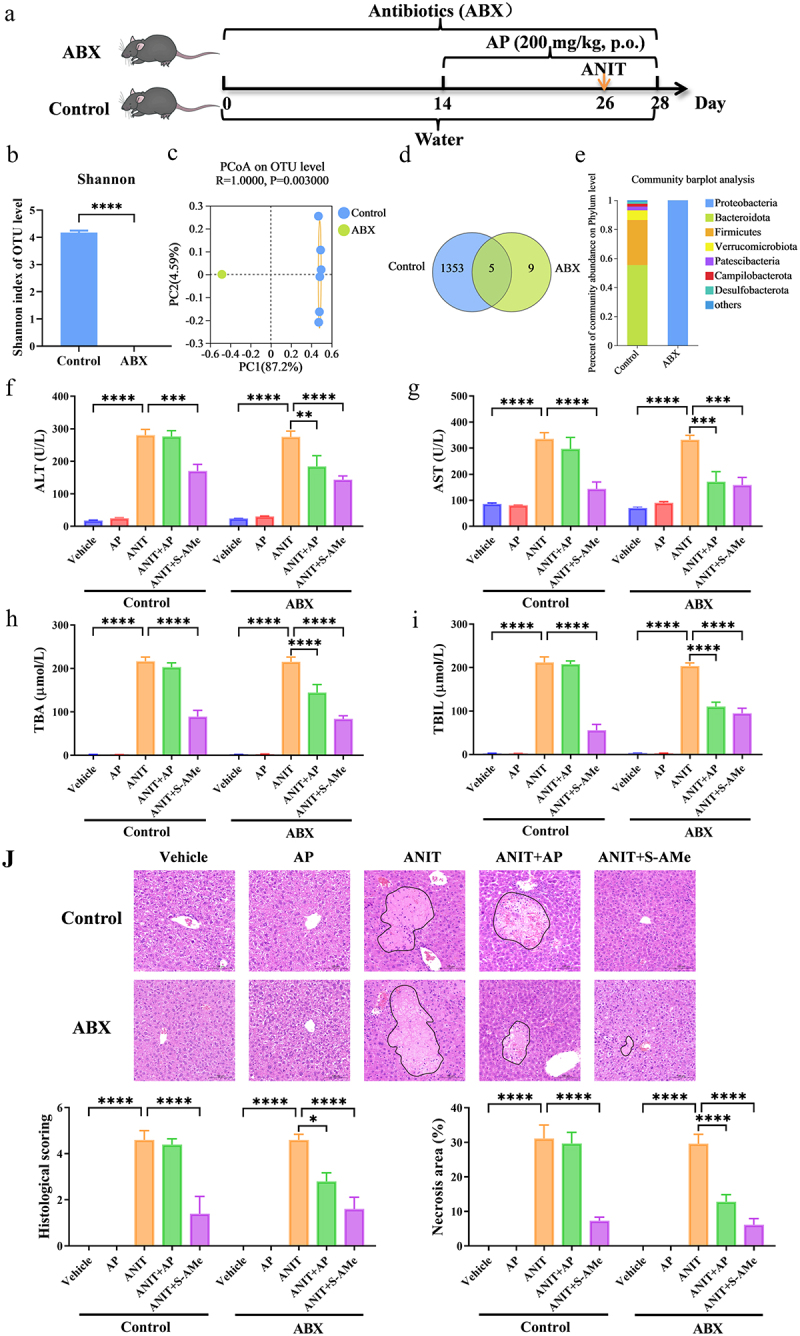
Gut microbiota depletion enhances the anti-cholestatic effect of AP. (a) Schematic representation of the procedure for administering antibiotics (ABX) and AP treatment in mice. (b) α-diversity analysis using the Shannon index at the operational taxonomic unit (OTU) level following 16S rRNA sequencing on fecal samples obtained from Control and ABX-treated mice (*n* = 6 per group). (c) β-diversity analysis using principal coordinates analysis (PCoA) on OTU level (*n* = 6 per group). (d) Number of shared OTUs between Control and ABX groups (*n* = 6 per group). (e) Percentage of community on the phylum level between Control and ABX groups (*n* = 6 per group). (f–i) Plasma levels of ALT, AST, TBA, and TBIL (*n* = 8 per group). (j) Representative H&E staining of the liver (magnification: 200×) and quantitative analysis of H&E staining score and necrosis area of the liver (*n* = 5 per group). Data are presented as mean ± SEM. *p < .05, **p < .01, ***p < .001, and ****p < 0.0001.

To investigate the impact of gut microbiota on the anti-cholestatic effect of AP, the anti-cholestatic effect of AP (200 mg/kg, p.o.) was compared between the Control and ABX mice (Figure 1(a)). A significant reduction in both the quantity and diversity of gut microbiota was noted following ABX treatment (Figure 1(b-e) and Supplementary Figure S1), indicating a substantial depletion of the gut microbiome under the influence of ABX. An intrahepatic cholestasis model was induced by ANIT, as demonstrated by the increase in key biochemical indicators (ALT, AST, TBA and TBIL) in plasma, along with the presence of hepatic necrosis and inflammatory cell infiltration (Figure 1(f-j)). In Control mice with intact intestinal flora, oral administration of AP at a dosage of 200 mg/kg showed a weak protective effect against ANIT-induced liver injury (Figure 1(f-j)). Conversely, in ABX mice where intestinal flora were depleted, AP at the same dosage markedly ameliorated ANIT-induced elevation of plasma biochemical indicators, liver cell necrosis, and hepatic inflammatory cell infiltration (Figure 1(f-j)). These findings suggest that gut microbiota impairs the anti-cholestatic effect of AP.

### 3.2. C-sulfonate metabolism mediated by gut microbiota impairs the anti-cholestatic effect of AP

**Figure 2.**
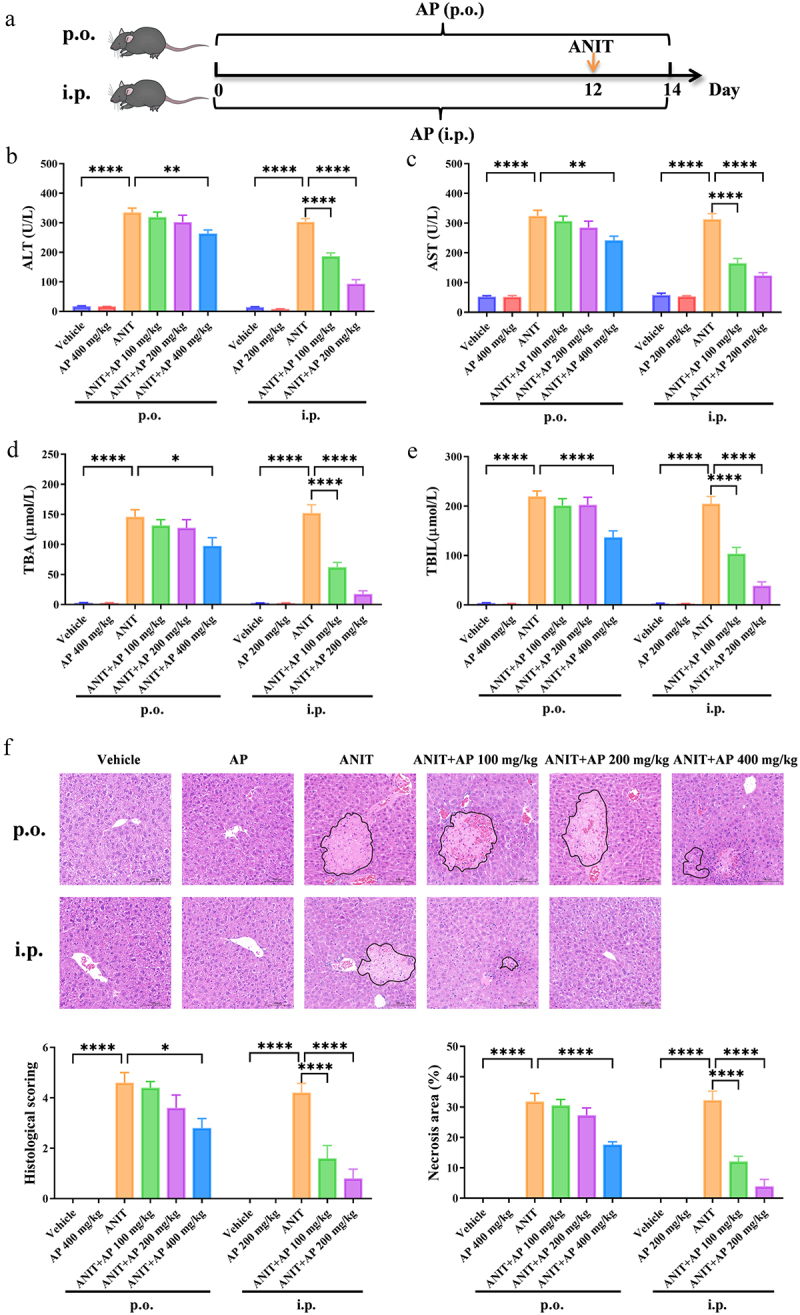
Intestinal C-sulfonate metabolism impairs the anti-cholestatic effect of AP. (a) Schematic diagram illustrating oral (p.o.) and intraperitoneal (i.p.) administration of AP at various dosages in mice. (b–e) Plasma levels of ALT, AST, TBA, and TBIL (*n* = 6 per group). (f) Representative H&E staining of the liver (magnification: 200×) and quantitative analysis of H&E staining score and necrosis area of the liver (*n* = 5 per group). Data are presented as mean ± SEM. *p < .05, **p < .01, and ****p < .0001.

To investigate the influence of C-sulfonate metabolism on the anti-cholestatic effect of AP, we administered AP to mice via oral (p.o.) and intraperitoneal (i.p.) routes (Figure 2(a)). Compared to the ANIT group, oral treatment with AP at low dosage of 100 or 200 mg/kg showed week hepatoprotective effect, while at a high dosage of 400 mg/kg showed significant efficacy (Figure 2(b-f)). In contrast, intraperitoneal treatment with AP at low dosage of 100 mg/kg or 200 mg/kg both exhibited a superior anti-cholestatic effect, significantly reducing the severity of hepatic damage (Figure 2(b-f)). These results strongly indicate that intraperitoneal administration of AP has a more potent anti-cholestatic effect compared to oral administration. This suggests that intestinal C-sulfonate metabolism significantly weakens the anti-cholestatic efficacy of AP.

**Figure 3.**
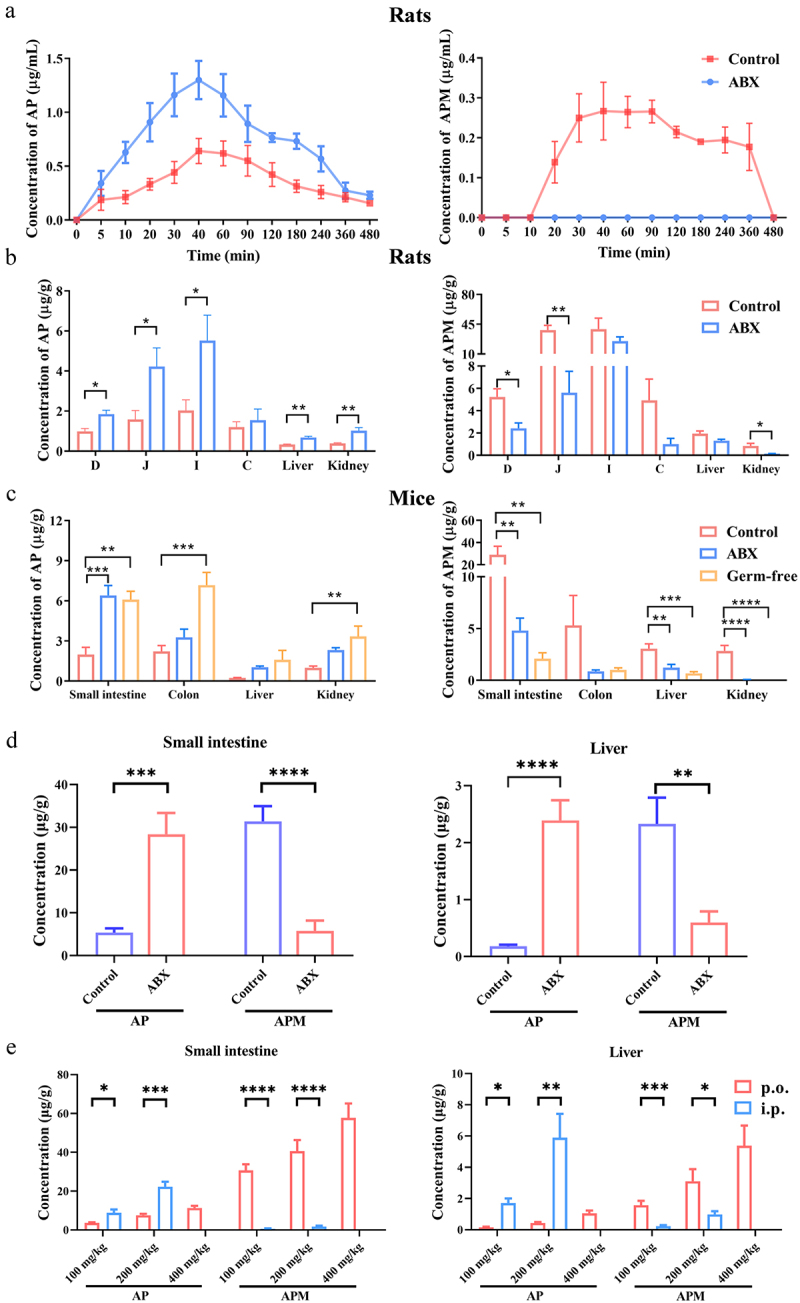
Gut microbiota mediates the C-sulfonate metabolism of AP. (a) Plasma pharmacokinetics of AP and APM following oral administration of AP (120 mg/kg) in rats (*n* = 4 per group). (b) Distribution of AP and APM in the duodenum (d), jejunum (j), ileum (i), colon (c), liver, and kidneys following oral administration of AP (120 mg/kg) in rats (*n* = 4 per group). (c) Distribution of AP and APM in the small intestine, colon, liver, and kidneys following oral administration of AP (100 mg/kg) in mice (*n* = 5 per group). (d) Concentrations of AP and APM in the small intestine and liver following AP treatment (200 mg/kg) in Control and ABX-treated mice (*n* = 8 per group). (e) Concentrations of AP and APM in the small intestine and liver following AP treatment at different dosages via oral (p.o.) or intraperitoneal (i.p.) administration routes in mice (*n* = 6 per group). Data are presented as mean ± SEM. *p < .05, **p < .01, ***p < .001, and ****p < .0001.

To explore the impact of gut microbiota on the C-sulfonate metabolism of AP, we employed ABX-treated rat, ABX-treated mouse and germ-free mouse models for comprehensive pharmacokinetic and tissue distribution analyses of AP. In ABX-treated rats, elevated levels of AP and reduced levels of APM were observed in plasma and tissues (Figure 3(a-b)), leading to a substantial 70.11% enhancement in the relative oral bioavailability of AP (Table 1). Similarly, in ABX-treated mice, the concentrations of AP and APM were notably increased and decreased, respectively, suggesting a reduction in the C-sulfonate metabolism of AP in mice with depleted gut microbiota (Figure 3(c)). Notably, in germ-free mice, the C-sulfonate metabolism of AP was also suppressed, consistent with the findings from ABX-treated rats and mice (Figure 3(c)). We further determined the concentrations of AP and APM in the small intestine and liver of Control and ABX-treated mice from previous pharmacological experiments and found that following ABX treatment, the concentration of AP significantly increased in the intestine and liver, while the level of its metabolite APM decreased significantly (Figure 3(d)). Additionally, in comparative pharmacological experiments of AP between oral administration and intraperitoneal injection, when AP exhibits better efficacy, its concentration in the small intestine and liver was correspondingly higher, while the concentration of its metabolite APM was lower (Figure 3(e)). Furthermore, our results indicated that the generation of the C-sulfonate metabolite was unaffected by the involvement of sulfotransferases (SULTs), as evidenced by the absence of detectable production of the metabolite APM in the classical incubation system of SULT-mediated metabolism (Suplementary Figure S2). Taken together, these findings convincingly establish the substantial role of gut microbiota in the C-sulfonate metabolism of AP, consequently diminishing the bioavailability and anti-cholestatic effect of AP.

**Table 1.** Plasma pharmacokinetics of AP following oral gavage administration at a dose of 120 mg/kg in rats from the Control and ABX groups. The presented values are the mean ± SD (*n* = 4 per group). *p < 0.05, **p < 0.01, compared to Control group.

Parameters	Units	Control	ABX
*T*_max_	min	50.00 ± 11.55	42.50 ± 12.58
*C*_max_	μg/mL	0.69 ± 0.19	1.33 ± 0.25**
*t*_1/2_	min	183.94 ± 55.70	138.82 ± 44.04
AUC_0-t_	mg/L*min	141.73 ± 28.80	265.51 ± 63.63*
AUC_0-∞_	mg/L*min	180.09 ± 42.46	306.35 ± 95.27
*F*_r_ (%)			170.11

### 3.3. *D. piger*-derived APS reductase mediates the C-sulfonate metabolism of AP

Our previous study demonstrated that large amounts of C-sulfonate metabolite APM were generated when AP was incubated with rat duodenal perfusate.^22^ Furthermore, we observed continued APM generation even after subjecting the duodenal perfusate to filtration and ultrafiltration (Suplementary Figure S3(a-b)). However, APM was not detectable when the perfusate underwent autoclaving treatment (Supplementary Figure S3(c)), coinciding with a significant reduction in the HSO_3_^−^ content of the perfusate (Supplementary Figure S3(d)). Remarkably, the addition of HSO_3_^−^ to the autoclaved perfusate resulted in the production of APM (Supplementary Figure S3(e)). These findings indicate that HSO_3_^−^ is an essential ligand for the C-sulfonate metabolism of AP, independent of host-derived enzymes participation.

**Figure 4.**
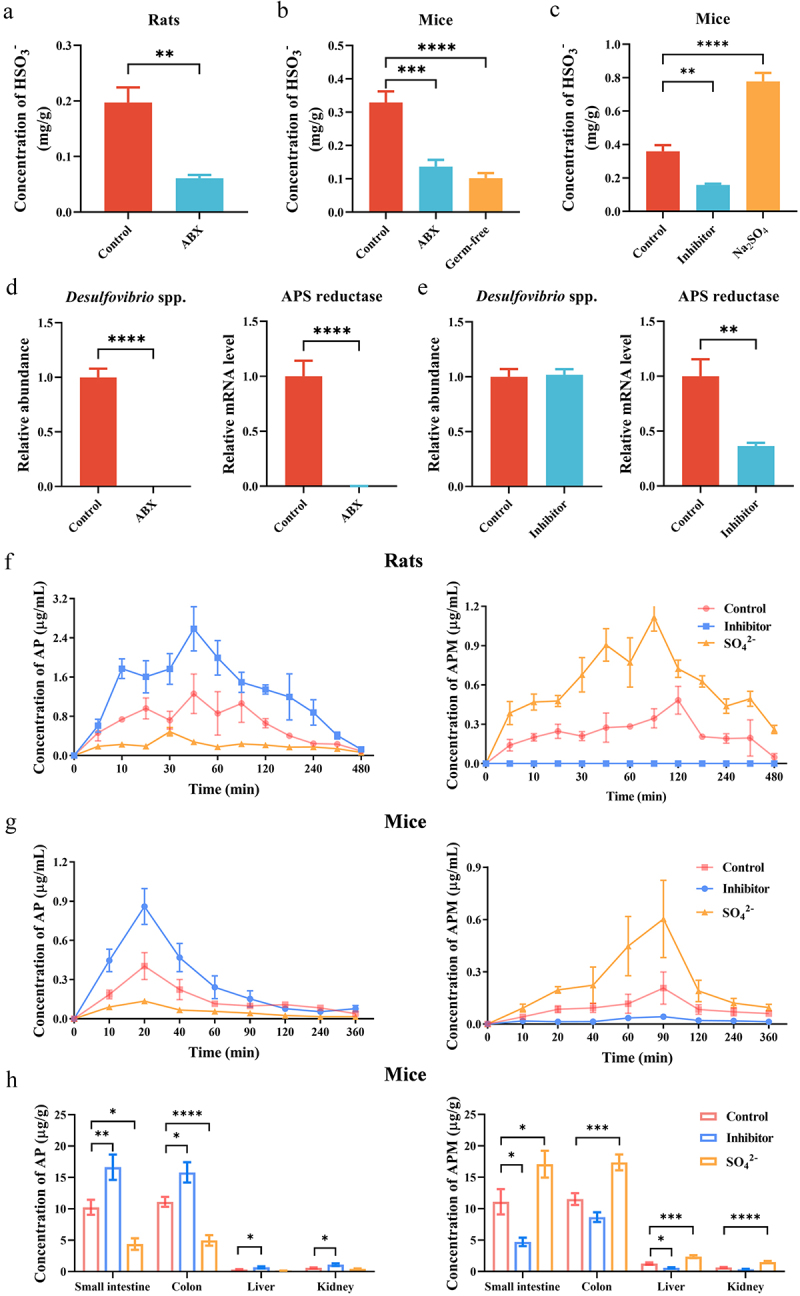
Gut microbiota-derived APS reductase mediates the C-sulfonate metabolism of AP. (a-b) Concentration of HSO_3_^−^ in the cecum of ABX-treated rats, ABX-treated mice and germ-free mice (*n* = 5 per group). (c) Concentration of HSO_3_^−^ in the cecum after oral administration of APS reductase inhibitor and Na_2_SO_4_ in mice (*n* = 4 per group). (d) Relative expression of *Desulfovibrio* spp. and APS reductase in the feces of Control and ABX-treated mice by qPCR (*n* = 8 per group). (e) Relative expression of *Desulfovibrio* spp. and APS reductase in the feces of mice after APS reductase inhibitor treatment by qPCR (*n* = 8 per group). (f) The effect of APS reductase inhibitor and Na_2_SO_4_ treatment on plasma pharmacokinetics of AP and APM after oral administration of AP (120 mg/kg) in rats (*n* = 4 for Control group and Inhibitor group, *n* = 5 for SO_4_^2-^ group). (g) The effect of APS reductase inhibitor and Na_2_SO_4_ treatment on plasma pharmacokinetics of AP and APM after oral administration of AP (100 mg/kg) in mice (*n* = 10 per group). (h) The effect of APS reductase inhibitor and Na_2_SO_4_ treatment on the distribution of AP and APM after oral administration of AP (100 mg/kg) in mice (*n* = 10 per group). Inhibitor refers to APS reductase inhibitor bromocriptine mesylate treatment and SO_4_^2-^ denotes Na_2_SO_4_ treatment. Data are presented as mean ± SEM. *p < .05, **p < .01, ***p < .001, and ****p < .0001.

To investigate whether HSO_3_^−^ originated from the intestine, we administered AP to mice and rats via the tail vein and orally, respectively. APM was undetectable in the intestine, liver, and kidneys after tail vein administration of AP, but a significant amount of APM was detected in all tissues after oral administration of AP (Supplementary Figure S4). This observation suggested that HSO_3_^−^ primarily originates from the intestine. HSO_3_^−^ is biosynthesized in vivo via two principal pathways: the host and the gut microbiota pathway. The host pathway involves the cysteine catabolic process, wherein cysteine is enzymatically converted into cysteine sulfinic acid by cysteine dioxygenase, subsequently undergoing decomposition to yield HSO_3_^−^.^48^ The gut microbiota pathway is the dissimilatory sulfate reduction route, wherein sulfate (SO_4_^2-^) undergoes reduction to form adenosine 5’-phosphosulfate (APS), and APS, in turn, serves as a precursor for the production of HSO_3_^−^ through the action of APS reductase, an enzyme produced by the gut microbiota (primarily by *Desulfovibrio* spp.).^49^ Our research findings demonstrated a significant decrease in HSO_3_^−^ content in the cecum of ABX-treated rats, ABX-treated mice, and germ-free mice (Figure 4(a-b)), accompanied by a notable reduction in the abundance of *Desulfovibrio* spp. and APS reductase in ABX-treated mice (Figure 4(d)). Furthermore, administration of the APS reductase inhibitor bromocriptine mesylate, which did not induce damage to organism in our study (Supplementary Figure S5), resulted in a decrease in HSO_3_^−^ content (Figure 4(c)) and APS reductase level (Figure 4(e)), while the administration of Na_2_SO_4_ led to a significant increase in HSO_3_^−^ content (Figure 4(c)). Inhibition of APS reductase activity by bromocriptine mesylate significantly suppressed the C-sulfonate metabolism of AP, resulting in a remarkable 117.24% increase in AP bioavailability in rats and a 47.48% increase in mice (Figure 4(f-g)and Tables 2,3), accompanied by elevated AP levels in mouse tissues (Figure 4(h)). Conversely, treatment of Na_2_SO_4_ enhanced the C-sulfonate metabolism of AP, leading to a reduction in its bioavailability by 49.30% in rats and 70.58% in mice (Figure 4(f-g) and Tables 2,3), coupled with decreased AP levels in mouse tissues (Figure 4(h)). Similarly, a substantial increase in AP C-sulfonate metabolism was observed following the addition of Na_2_SO_4_ in an in vivo intestinal perfusion assay conducted in rats (Supplementary Figure S6).

**Table 2.** Plasma pharmacokinetics of AP following oral gavage administration at a dose of 120 mg/kg in rats from the Control, Inhibitor and SO_4_^2-^ groups.

Parameters	Units	Control	Inhibitor	SO_4_^2-^
*T*_max_	min	30.00 ± 24.50	35.00 ± 10.00	32.00 ± 4.47
*C*_max_	μg/mL	1.42 ± 0.71	2.75 ± 0.71*	0.49 ± 0.19*
t_1/2_	min	122.32 ± 23.81	109.32 ± 35.35	262.83 ± 194.05
AUC_0-t_	mg/L*min	206.23 ± 59.18	451.39 ± 124.08*	81.66 ± 20.02**
AUC_0-∞_	mg/L*min	221.37 ± 58.26	480.90 ± 120.03**	112.23 ± 35.42*
*F*_r_ (%)			217.24	50.70

The presented values are the mean ± SD (*n* = 4 for Control group and Inhibitor group, *n* = 5 for SO_4_^2-^ group). *p < .05, **p < .01, compared to control group.

**Table 3.** Plasma pharmacokinetics of AP following oral gavage administration at a dose of 100 mg/kg in mice from the Control, Inhibitor and SO_4_^2-^ groups.

Parameters	Units	Control	Inhibitor	SO_4_^2-^
*T*_max_	min	32.00 ± 32.59	27.00 ± 14.94	17.00 ± 4.83
*C*_max_	μg/mL	0.50 ± 0.33	0.97 ± 0.37**	0.142 ± 0.03**
t_1/2_	min	145.97 ± 67.38	145.86 ± 86.38	133.19 ± 104.25
AUC_0-t_	mg/L*min	38.78 ± 9.13	54.34 ± 19.38*	11.93 ± 3.71****
AUC_0-∞_	mg/L*min	46.29 ± 14.77	68.27 ± 30.94	13.62 ± 4.03****
*F*_r_ (%)			147.48	29.42

The presented values are the mean ± SD (*n* = 10 per group). *p < .05, **p < .01, ****p < .0001, compared to control group.

**Figure 5.**
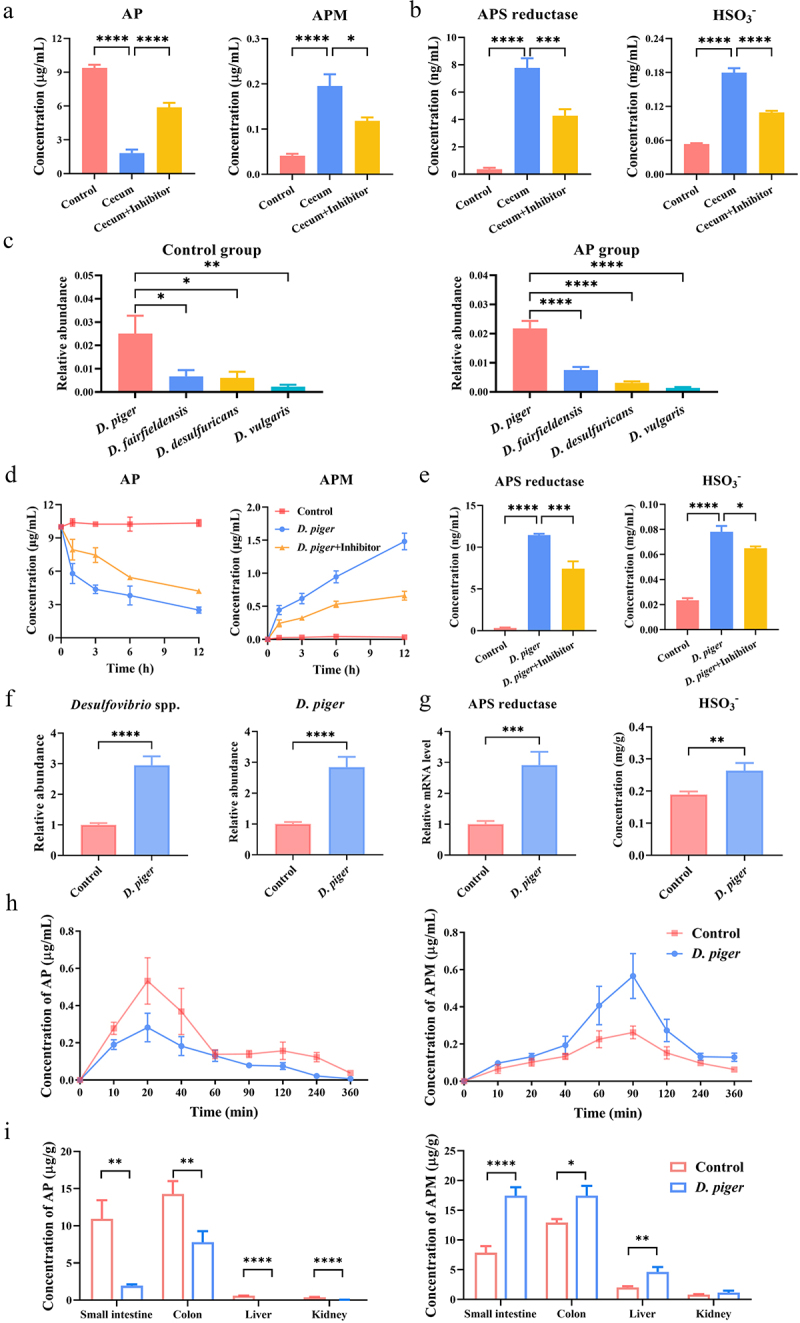
*D. piger*-derived APS reductase mediates the C-sulfonate metabolism of AP. (a) Concentrations of AP and APM following co-cultured of AP with cecum samples from mice in the absence or presence of APS reductase inhibitor (*n* = 5 per group). (b) Concentrations of APS reductase and HSO_3_^−^ following co-cultured of AP with cecum samples from mice in the absence or presence of APS reductase inhibitor (*n* = 5 per group). (c) Relative abundance of four *Desulfovibrio* species obtained from shotgun metagenomic sequencing analysis of cecum samples in Control and AP-treated mice (*n* = 8 per group). (d) Concentrations of AP and APM following co-cultured of AP with *D. piger* in the absence or presence of APS reductase inhibitor (*n* = 5 per group). (e) Concentrations of APS reductase and HSO_3_^−^ following co-cultured of AP with *D. piger* in the absence or presence of APS reductase inhibitor (*n* = 5 per group). (f) Relative abundance of *Desulfovibrio* spp. and *D. piger* in mouse fecal samples post-colonization of *D. piger* using qPCR (*n* = 12 per group). (g) Relative mRNA level of APS reductase and concentration of HSO_3_^−^ in mouse fecal samples post-colonization of *D. piger* (*n* = 12 per group). (h) The effect of *D. piger* treatment on plasma pharmacokinetics of AP and APM after oral administration of AP (100 mg/kg) in mice (*n* = 12 per group). (i) The effect of *D. piger* treatment on the distribution of AP and APM after oral administration of AP (100 mg/kg) in mice (*n* = 12 per group). Data are presented as mean ± SEM. *p < .05, **p < .01, ***p < .001, and ****p < .0001.

In order to enhance the understanding of the cause–effect relationship between *Desulfovibrio* spp., AP’s C-sulfonate metabolism and bioavailability, we carried out a series of experiments. For experiments involving the co-culturing of AP with cecum samples from mice, the results unveiled a notable conversion of AP into APM by the intestinal flora of mice (Figure 5(a)). This conversion was effectively impeded upon the addition of an APS reductase inhibitor (Figure 5(a)). Moreover, concomitant with this inhibition, there was a significant decrease in the concentrations of APS reductase and HSO_3_^−^ (Figure 5(b)), suggesting that APS reductase produced by gut microbiota facilitates the C-sulfonate metabolism of AP. To further clarify the specific *Desulfovibrio* species responsible for APS reductase production and involvement in AP’s C-sulfonate metabolism, we conducted shotgun metagenomics analysis. We discovered four predominant *Desulfovibrio* species (*D. piger*, *D. fairfieldensis*, *D.* *desulfuricans* and *D.* *vulgaris*) in both Control and AP-treated mice. Subsequent analysis revealed that *D. piger* exhibited the highest abundance in both groups (Figure 5(c)). Notably, *D. piger* is one of the most prevalent and abundant *Desulfovibrio* species in the human body.^50–52^ Consequently, we proceeded to co-culture AP with *D. piger* and observed its time-dependent ability to metabolize AP into APM in vitro through the production of APS reductase and HSO_3_^−^. This activity was significantly inhibited by an APS reductase inhibitor (Figure 5(d-e)). To further validate the role of *D. piger*, we successfully transplanted it into mice. This resulted in a significant increase in the relative abundance of APS reductase and HSO_3_^−^ concentration (Figure 5(f-g)), leading to a marked enhancement in the C-sulfonate metabolism of AP and subsequently reducing its bioavailability by 60.11% (Figure 5(h-i), Table 4). Furthermore, following colonization of *D. piger* in mice, there was a notable reduction in AP concentration in tissues such as the liver, along with an increase in the concentration of APM (Figure 5(i)).

**Table 4.** Plasma pharmacokinetics of AP following oral gavage administration at a dose of 100 mg/kg in mice from the Control and *D. piger* groups.

Parameters	Units	Control	D. piger
*T*_max_	min	41.67 ± 39.27	35.00 ± 31.77
*C*_max_	μg/mL	0.76 ± 0.44	0.36 ± 0.28*
*t*_1/2_	min	135.90 ± 65.22	77.18 ± 38.61*
AUC_0-t_	mg/L*min	54.56 ± 13.99	22.45 ± 7.65****
AUC_0-∞_	mg/L*min	61.80 ± 15.00	24.65 ± 7.04****
*F*_r_ (%)			39.89

The presented values are the mean ± SD (*n* = 12 per group). *p < .05, ****p < .0001, compared to Control group.

These collective findings underscore the crucial role of APS reductase, produced by *D. piger*, in mediating the C-sulfonate metabolism of AP and modulating its bioavailability.

### 3.4. Inhibition of APS reductase enhances the anti-cholestatic effect of AP

**Figure 6.**
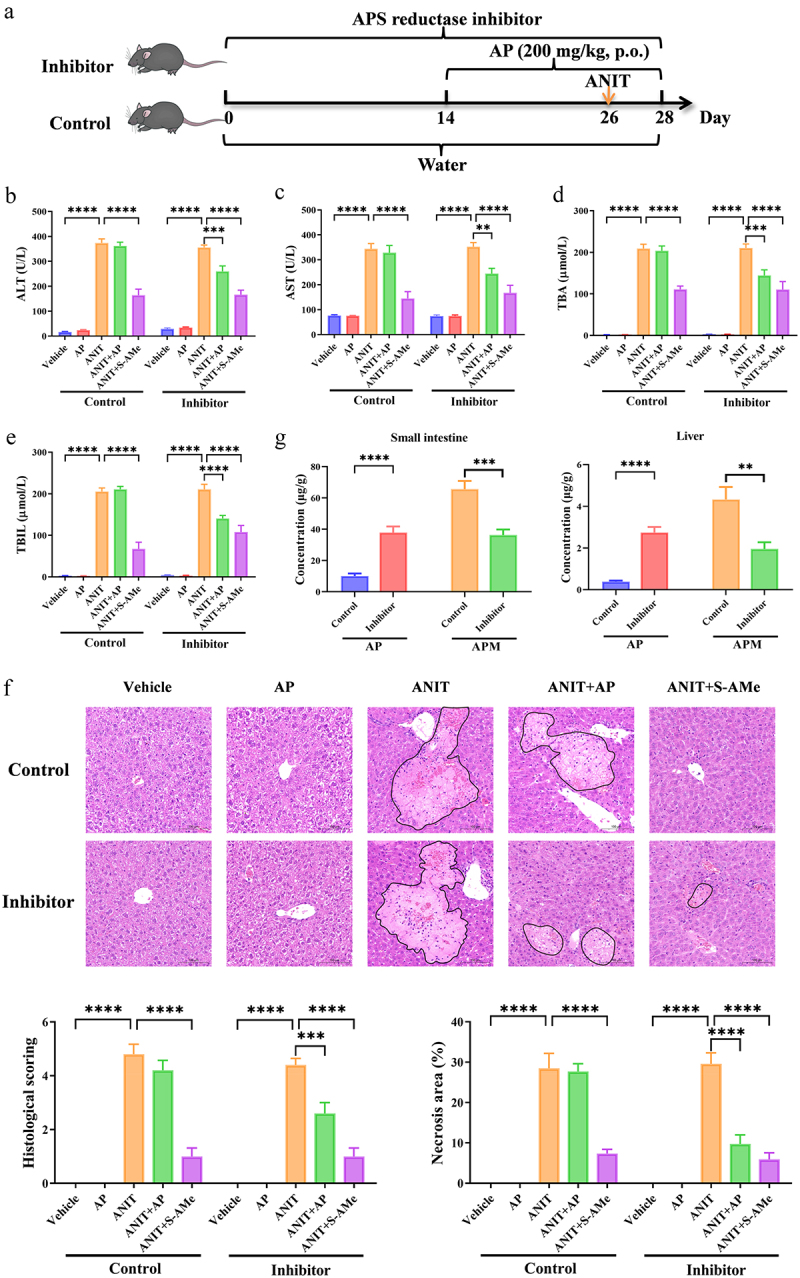
APS reductase inhibitor enhances the anti-cholestasis effect of AP. (a) Schematic representation of the treatment procedure involving APS reductase inhibitor (bromocriptine mesylate, 20 mg/kg, p.o.) and AP (200 mg/kg, p.o.) in mice. (b–e) Plasma levels of ALT, AST, TBA, and TBIL (*n* = 8 per group). (f) Representative H&E staining of the liver (magnification: 200×) and quantitative analysis of H&E staining score and necrosis area of the liver (*n* = 5 per group). (g) Concentrations of AP and APM in the small intestine and liver following AP treatment in the control and APS reductase inhibitor-treated mice (*n* = 8 per group). Data are presented as mean ± SEM. **p < .01, ***p < .001, and ****p < .0001.

To ascertain the impact of APS reductase-mediated C-sulfonate metabolism on the anti-cholestatic efficacy of AP, we conducted a treatment study in mice wherein AP was administered in combination with APS reductase inhibitor bromocriptine mesylate (Figure 6(a)). Our research findings revealed that the administration of AP (200 mg/kg, p.o.) alone did not exhibit significant alleviation of ANIT-induced cholestatic liver injury (Figure 6(b-f)). However, when AP was administered in combination with APS reductase inhibitor, a notable amelioration of liver injury as observed (Figure 6(b-f)). Furthermore, the APS reductase inhibitor effectively suppressed the C-sulfonate metabolism of AP, resulting in an increase in AP exposure and a corresponding decrease in APM within the small intestine and liver (Figure 6(g)). Based on these results, we conclude that the anti-cholestatic effect of AP can be enhanced through inhibition of APS reductase, which mediates the C-sulfonate metabolism of AP. Consequently, the co-administration of AP with APS reductase inhibitors holds promise as a robust and efficacious therapeutic strategy for cholestasis.

### 3.5. AP exerts anti-cholestatic effects via activating FXR-related pathway

**Figure 7.**
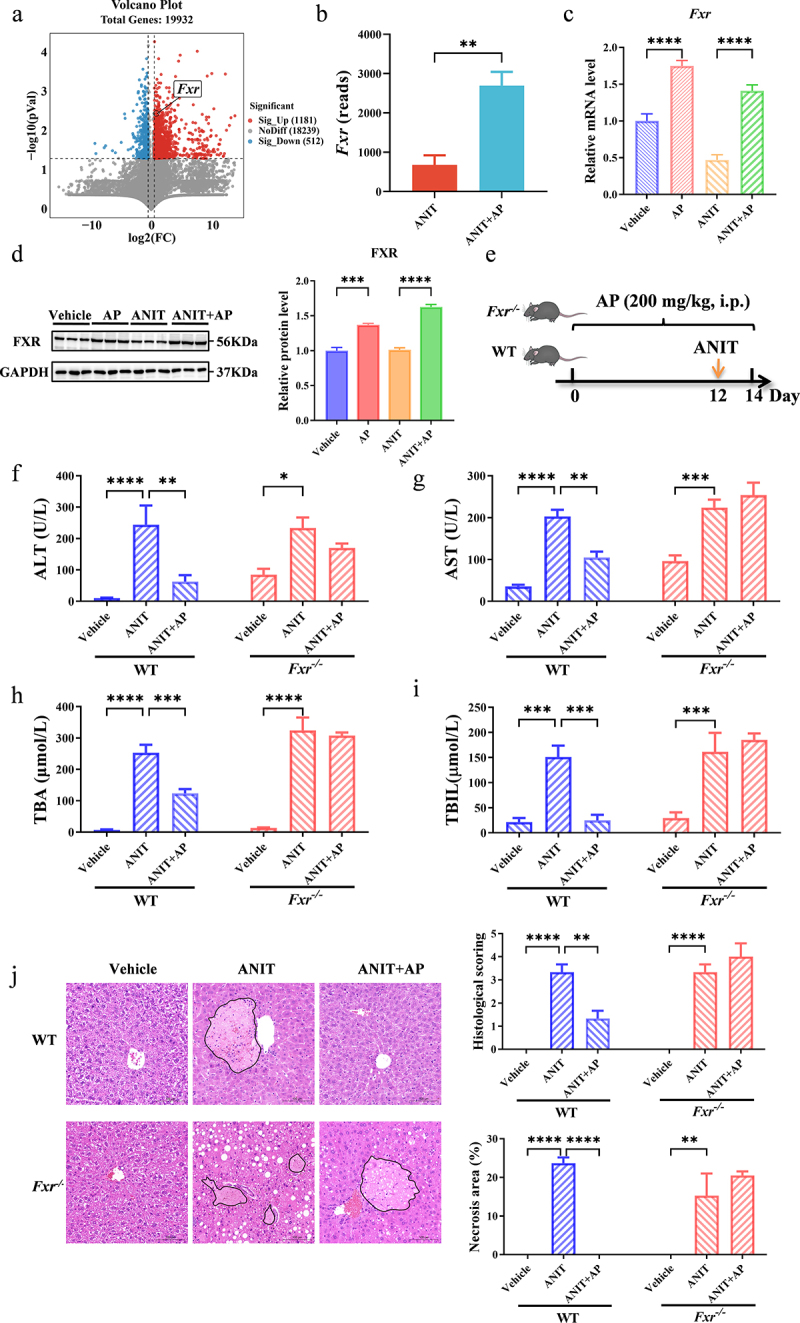
AP exerts anti-cholestatic effects by agonizing FXR. (a) Volcano plot depicting the differentially expressed genes between the ANIT group and the ANIT+AP group, based on RNA sequencing analysis conducted on liver samples from mice treated with ANIT and ANIT+AP (*n* = 3 per group). (b) The read counts of *Fxr* obtained from RNA sequencing analysis on liver samples collected from mice treated with ANIT and ANIT+AP (*n* = 3 per group). (c) Relative mRNA level of *Fxr* in the liver of mice as determined by qPCR (*n* = 5 per group). (d) Relative protein level of FXR in the liver of mice as assessed by western blot analysis (*n* = 3 per group). (e) Schematic diagram illustrating administration of AP in WT and *Fxr^−/−^* ice. (f-i) Plasma levels of ALT, AST, TBA, and TBIL (*n* = 5 per group). (j) Representative H&E staining of the liver (magnification: 200×) and quantitative analysis of H&E staining score and necrosis area of the liver (*n* = 3 per group). Data are presented as mean ± SEM. *p < .05, **p < .01, ***p < .001, and ****p < .0001.

**Figure 8.**
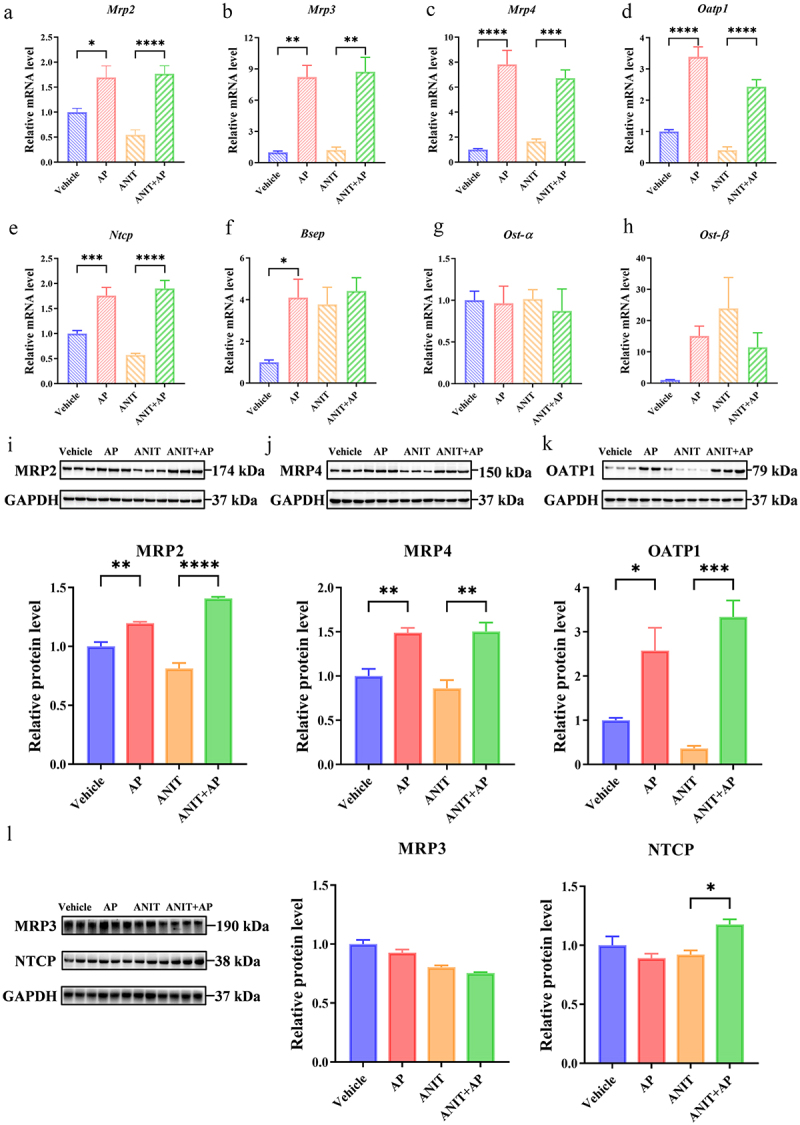
AP upregulates the relative mRNA and protein levels of FXR downstream bile acid transporters. (a–h) Relative mRNA levels of *Mrp2, Mrp3, Mrp4, Oatp1, Ntcp, Bsep, Ost-α* and *Ost-β* in the liver of mice by qPCR (*n* = 5 per group). (i–l) Relative protein levels of MRP2, MRP3, MRP4, OATP1 and NTCP in the liver of mice by western blot analysis (*n* = 3 per group). Data are presented as mean ± SEM. *p < .05, **p < .01, ***p < .001, and ****p < .0001.

To further investigate the mechanism by which AP alleviates cholestasis, we performed RNA-sequencing analysis to identify the potential targets and pathways of AP for the treatment of cholestasis. Previous studies have shown that AP exerts its anticholestatic effects mainly through the NF-κB and JNK/MAPK pathways.^15,53,54^ However, our analysis revealed no significant differences in the clustering of both NF-κB pathway genes (*Vcam-1, Il-4, Il-6, Tnf,* and *Ptgs2*) and JNK/MAPK pathway genes (*Tnf, Il-1β, Timp-1, Mmp-2, Bax,* and *Bcl-2*) (Supplementary Figure S7). Nevertheless, our findings showed a significant upregulation in the expression of *Fxr* (Nr1h4) (Figure 7(a-b)). Consistently, both qPCR and Western blot analyses confirmed the upregulation of FXR expression after AP treatment (Figure 7(c-d)). FXR plays a crucial role in regulating bile acid transporters, thus exerting an important function in anti-cholestatic processes.^41,55,56^ Subsequently, we employed both WT and *Fxr*^*-/-*^ mice in our study to test whether AP exerts anti-cholestatic effects via activating FXR-related pathway (Figure 7(e)). In WT mice, AP exhibited a superior anti-cholestatic effect (Figure 7(f-j)). However, in *Fxr*^*-/-*^ mice, the beneficial effect of AP was completely abolished at the same dosage (), highlighting the dependence of AP’s anti-cholestatic effect on FXR (Figure 7(f-j)). Furthermore, we observed that AP heightened the expression of FXR downstream bile acid transporters (*Mrp2*, *Mrp3*, *Mrp4*, *Oatp1*, and *Ntcp*, except for *Bsep*, *Ost-α* and *Ost-β*) (Figure 8(a-h)). Western blot analysis showed that the expression of MRP2, MRP4, and OATP1 was consistent with qPCR analysis (Figure 8(i-l)). In conclusion, these findings suggest that AP exerts its anti-cholestatic effect by activating FXR and thereby upregulating MRP2 and MRP4.

## 4. Discussion

Cholestatic liver injury, characterized by the accumulation of toxic bile acids in the liver, presents a significant challenge in clinical management due to the lack of effective therapeutic options.^57,58^ Despite previous studies demonstrating the protective potential of AP against cholestatic liver injury,^15,16^ its efficacy may be hindered by low bioavailability.^22^ Our study focused on exploring the underlying mechanisms of AP’s low bioavailability, shedding light on the critical role played by gut microbiota in modulating its pharmacokinetics and anti-cholestatic effect.

One of the primary findings of our study, utilizing pseudo- and germ-free models, was the discovery that gut microbiota significantly influences the metabolism of AP, resulting in the formation of a C-sulfonate metabolite, APM. This transformative metabolic pathway is mediated by a specific gut microbial enzyme, APS reductase, which catalyzes the reduction of SO_4_^2-^ to HSO_3_^−^. HSO_3_^−^ subsequently interacts with AP, targeting its C=C unsaturated double bond, thereby leading to the formation of the C-sulfonate metabolite. It is important to note that C-sulfonate metabolism constitutes a distinctive pathway, separate from the O- and N-sulfonate metabolism pathway governed by SULTs.^29,30^ Significantly, this study represents the first documentation of gut microbiota, especially *D. piger*-derived APS reductase’s pivotal involvement in directing the C-sulfonate metabolism of AP, thereby introducing a novel dimension to the already intricate network of drug metabolic pathways. In our study, we found that clearing gut microbiota or inhibiting APS reductase can significantly improve the bioavailability of AP. By depleting gut microbiota using ABX, the relative bioavailability of AP increased by 70%, while inhibiting APS reductase activity led to a relative bioavailability increase of 117% in rats. It is noteworthy that a multitude of pharmaceutical agents undergo metabolism not only by the host but also by the gut microbiota. This complexity is exemplified by various drugs, such as acarbose,^38^ aspirin,^36^ and levodopa,^59^ indicating the intricate interplay of drug metabolism within the gut microbiota. Notably, HSO_3_^−^ is generated in vivo by both the host and the intestinal flora.^48,60^ While our research elucidated the vital role of intestinal flora-produced HSO_3_^−^ in AP metabolism, it raises the intriguing question of whether host-derived HSO_3_^−^ plays a role in this process, warranting further exploration and investigation.

Our study also demonstrated a remarkable enhancement in AP’s anti-cholestatic effect upon inhibition of APS reductase produced by *Desulfovibrio* spp. (particularly *D. piger*). *D. piger* is one of the most common and abundant *Desulfovibrio* species in the intestinal tract of both mice and humans.^50^ Our investigation revealed that *D. piger* metabolizes AP through the production of APS reductase in vitro and in vivo, thereby impeding the bioavailability of AP. Therefore, it is highly probable that *D. piger* affects AP’s C-sulfonate metabolism in the human body. This finding suggests a potential strategy for optimizing the therapeutic application of AP in patients suffering from cholestatic liver injury. APS reductase, predominantly produced by sulfate-reducing bacteria including *Desulfovibrio* spp.,^49^ serves as a pivotal enzyme within the sulfate reduction metabolic pathway, playing a vital role for the growth and development of both plants and animals.^61^ It is notable that APS reductase has garnered attention as a validated target for the development of novel antitubercular agents.^62^ Several compounds with inhibitory effects on APS reductase have been investigated biologically, including bromocriptine, flupirtine, and mexiletine.^63,64^ These compounds have already found clinical utility in the treatment of conditions such as Parkinson’s disease, acute pain, and migraines, respectively^65–67^ In our study, the co-administration of AP with the APS reductase inhibitor bromocriptine significantly enhanced the anti-cholestatic effect of AP. Thus, combining these inhibitors with AP holds substantial promise for enhancing the clinical efficacy of AP in the treatment of cholestatic liver injury. Additionally, strategies aimed at modifying gut microbiota composition and subsequently reducing APS reductase activity will also enhance the clinical efficacy of AP in the treatment of cholestatic liver injury. These strategies involve utilizing probiotics like *Lactobacillus* and *Bifidobacterium*, consuming prebiotic-rich foods to promote beneficial bacteria growth, limiting sulfur intake to hinder sulfate-reducing bacteria, and in some cases, employing targeted antibiotic therapy.^68–71^ Our shotgun metagenomic analysis revealed that AP treatment did not induce significant alterations in the expression of APS reductase or inhibit the growth of APS reductase-producing gut microbiota, despite its ability to influence the abundance and composition of gut microbes (Supplementary Figure S8(a-f)). The Xiaoyanlidan formula, with AP as its quality control component, exhibits promising clinical efficacy in addressing cholestatic liver injury.^17,18^ Further investigation is warranted to ascertain whether specific components within it have the potential to inhibit APS reductase directly or impede the proliferation of APS reductase-producing bacterial strains.

Furthermore, our investigation provided intriguing insights into the mechanisms underlying the anti-cholestatic effects of AP. While previous studies suggested that AP exerted its anti-cholestatic effects primarily through the NF-κB and JNK/MAPK pathways,^15,53,54^ our transcriptomic results revealed no significant effect of AP on these pathways. Instead, we observed upregulation of FXR following AP treatment, confirmed by qPCR and WB analysis. Further, we compared the anti-cholestatic effects of AP in wild-type mice and FXR knockout mice, observing significant effects in wild-type mice but no protective effect in knockout mice. FXR, a nuclear receptor, acts as a key regulator of bile acid synthesis, transport, and metabolism.^55^ Specifically, FXR activation regulates the expression of bile acid transporters, such as BSEP, MRP2/3/4.^72^ BSEP and MRP2 facilitate the secretion of bile acids into bile, while MRP3 and MRP4 responsible for transporting bile acids out of hepatocytes into the bloodstream.^73^ We found that AP upregulated the gene and protein levels of MRP2 and MRP4 in our pharmacological model, consistent with a previous study.^15^ Our results also indicated that AP upregulated OATP1 expression, while the expression of OATP1 regulated by FXR may vary in different contexts.^74,75^ Traditional Chinese medicines like Yin Chen Hao Decoction and *Gardenia jasminoides* Ellis have demonstrated their ability to ameliorate cholestatic liver injury through FXR-mediated mechanisms.^76,77^ Currently, commercially available dosage forms of AP include tablets, capsules, and dripping pills. The inherent qualities of AP, such as its well-tolerated nature, high safety profile, absence of side effects or adverse reactions, and cost-effectiveness,^78^ position it as a potent therapeutic agent for cholestasis. Previous studies have underscored a close association between cholestatic diseases and the gut microbiota, with specific focus on *Enterococcus*, *Lactobacillus*, and *Escherichia* bacteria, which are deemed pivotal in their onset and progression.^79,80^ Nonetheless, our shotgun metagenomic analysis unveiled that AP did not suppress the expression of these bacteria (Supplementary Figure S8(g-i)), despite eliciting changes in the composition of the gut microbiota. However, whether AP exerts its anti-cholestatic effects by intervening with other bacteria requires further investigation.

**Figure 9.**
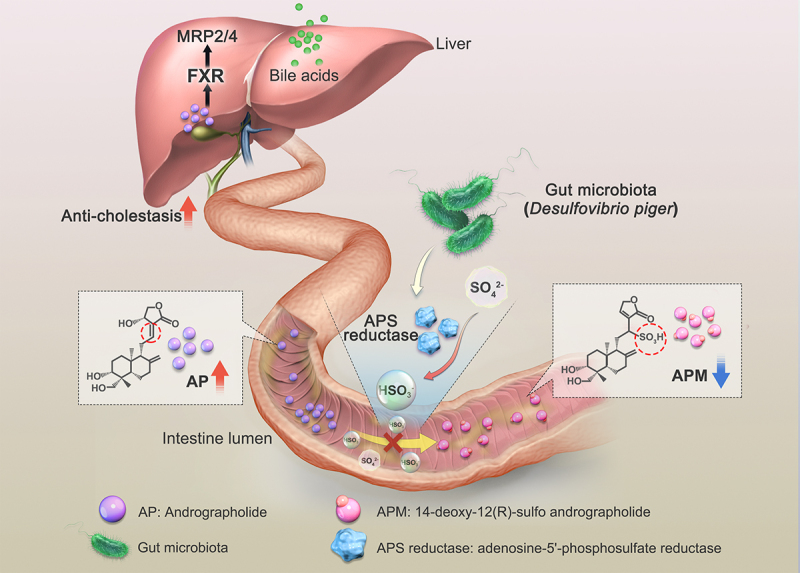
The APS reductase secreted by gut microbiota, particularly *Desulfovibrio piger,* mediates the C-sulfonate metabolism of AP, thereby impairing its bioavailability and anti-cholestatic efficacy.

In conclusion, our study has unraveled the intricate relationship between gut microbiota especially *Desulfovibrio piger* and the C-sulfonate metabolism of AP, shedding light on a novel pathway for optimizing its therapeutic effects in cholestatic liver injury (Figure 9). The identification of APS reductase as a key enzyme and the potential for its inhibition to enhance AP bioavailability hold promise for future drug development efforts. Furthermore, the engagement of FXR-mediated MRP2/4 in AP’s mechanism of action enhances our comprehension of its therapeutic potential. These findings offer valuable insights and open new avenues for improving the clinical application of AP as a promising treatment for cholestatic liver injury.

## 5. Conclusion

The C-sulfonate metabolism of AP is facilitated by APS reductase, which is produced by gut microbiota, particularly *Desulfovibrio piger*. Targeting APS reductase leads to in an increase of the bioavailability and anti-cholestatic efficacy of AP.

## Supplementary Material

Supplemental Material

## Data Availability

The 16S rRNA and shotgun metagenomic sequencing datasets are available in the NCBI SRA database under the accession numbers PRJNA1073304 and PRJNA1073453, respectively. Additionally, the RNA sequencing data have been deposited in the NCBI SRA database under the accession number PRJNA1073703. The remaining data are available within the article, Supplementary Materials, or Source data file.
