# Supplementary material for: Gut microbiota-mediated C-sulfonate metabolism impairs the bioavailability and anti-cholestatic efficacy of andrographolide
Source: Gut Microbes. 2024 Sep 12;16(1):2387402. doi: 10.1080/19490976.2024.2387402 (PMC11404609; doi:10.1080/19490976.2024.2387402)
Supplement: Supplemental Material [file KGMI_A_2387402_SM4329.zip › Supplementary materials (2).docx]

Gut microbiota-mediated C-sulfonate metabolism impairs the bioavailability and anti-cholestatic efficacy of andrographolide

Dafu Tang^a, 1^, Wanyu Hu^a, 1^, Bingxuan Fu^a^, Xiaojie Zhao^a^, Guoquan You^a^, Cong Xie^b^, Hong yu Wang^a^, Xueni Guo^a^, Qianbing Zhang^c^, Zhongqiu Liu^a, d, *^, Ling Ye^a, *^

^a^NMPA Key Laboratory for Research and Evaluation of Drug Metabolism & Guangdong Provincial Key Laboratory of New Drug Screening, School of Pharmaceutical Sciences, Southern Medical University, Guangzhou 510515, China.

^b^Clinical Pharmacy Center, Nanfang Hospital, Southern Medical University, Guangzhou 510515, China.

^c^Cancer Research Institute, School of Basic Medical Sciences, Southern Medical University, Guangzhou 510515, China.

^d^International Institute for Translational Chinese Medicine, Guangzhou University of Chinese Medicine, Guangzhou, Guangdong 510006, China.

^1^These authors contribute equally to this work.

^*^Corresponding authors:

Ling Ye, Ph.D. School of Pharmaceutical Sciences, Southern Medical University, Guangzhou, 510515, Guangdong, China. Tel: +86-20-61648597, E-mail: [yeling623@hotmail.com](mailto:yeling623@hotmail.com)

Zhongqiu Liu, Ph.D. International Institute for Translational Chinese Medicine, Guangzhou University of Chinese Medicine, Guangzhou, Guangdong 510006, China. E-mail: [liuzq@gzucm.edu.cn](mailto:yeling623@hotmail.com)


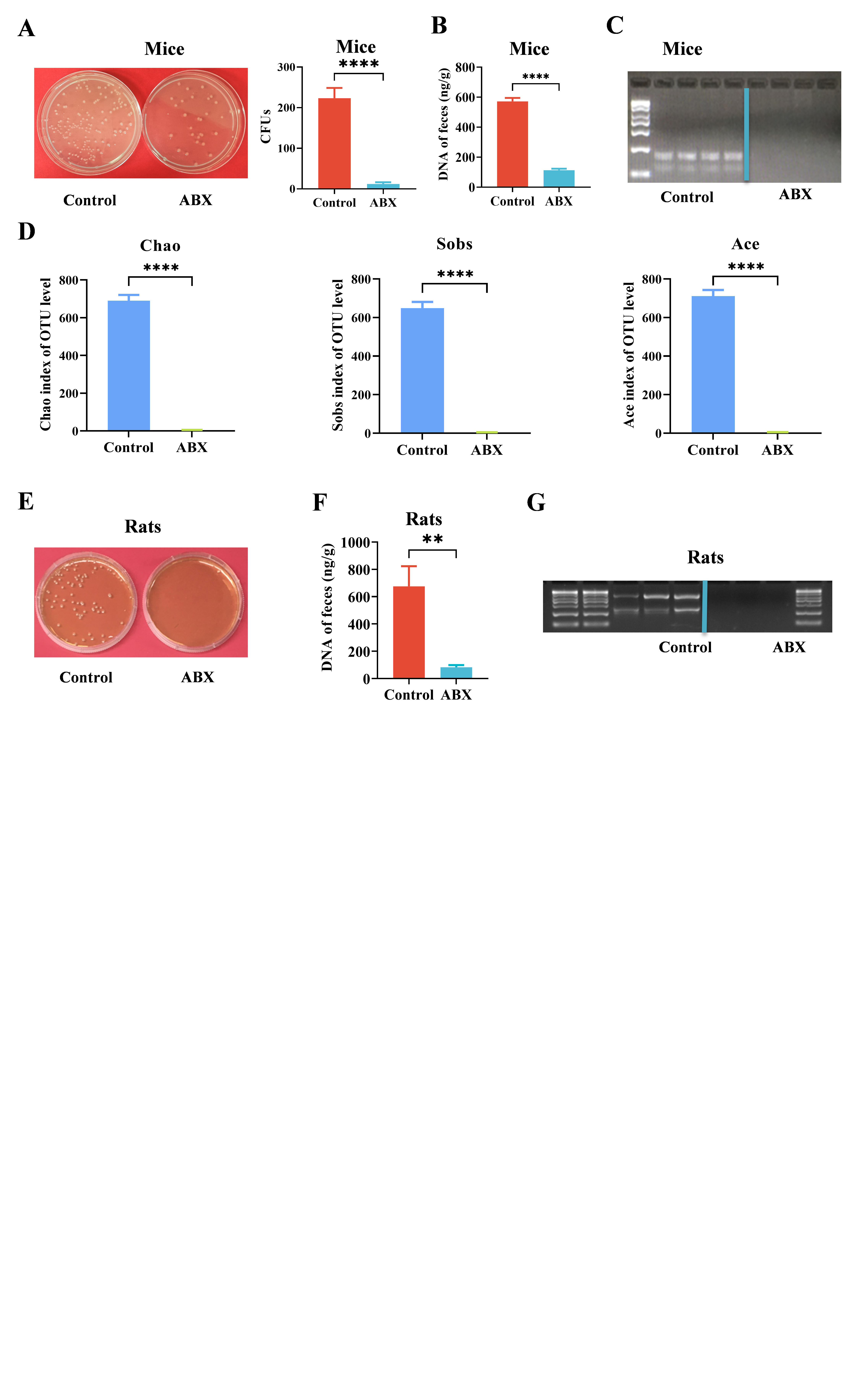


Supplementary Figure 1. Identification of the efficacy of antibiotic (ABX) in eliminating gut microbiota. (A) Cultivation and count of viable bacteria of mouse feces (n = 5 per group). (B) Bacterial DNA extracted from mouse feces (n = 8 per group). (C) PCR amplification bands of bacterial 16S rRNA genes extracted from mouse fecal samples using agarose gel electrophoresis (n = 4 per group). (D) α-diversity analysis using the Chao index, Sobs index, and Ace index at the operational taxonomic unit (OTU) level following 16S rRNA sequencing on fecal samples obtained from Control and ABX-treated mice (n = 6). (E) Cultivation of viable bacteria of rat feces. (F) Bacterial DNA extracted from rat feces (n = 4 per group). (G) PCR amplification bands of bacterial 16S rRNA genes extracted from rat feces using agarose gel electrophoresis (n = 3 per group). Data are presented as mean ± SEM. ***p* < 0.01, ****p* < 0.001 and *****p* < 0.0001.


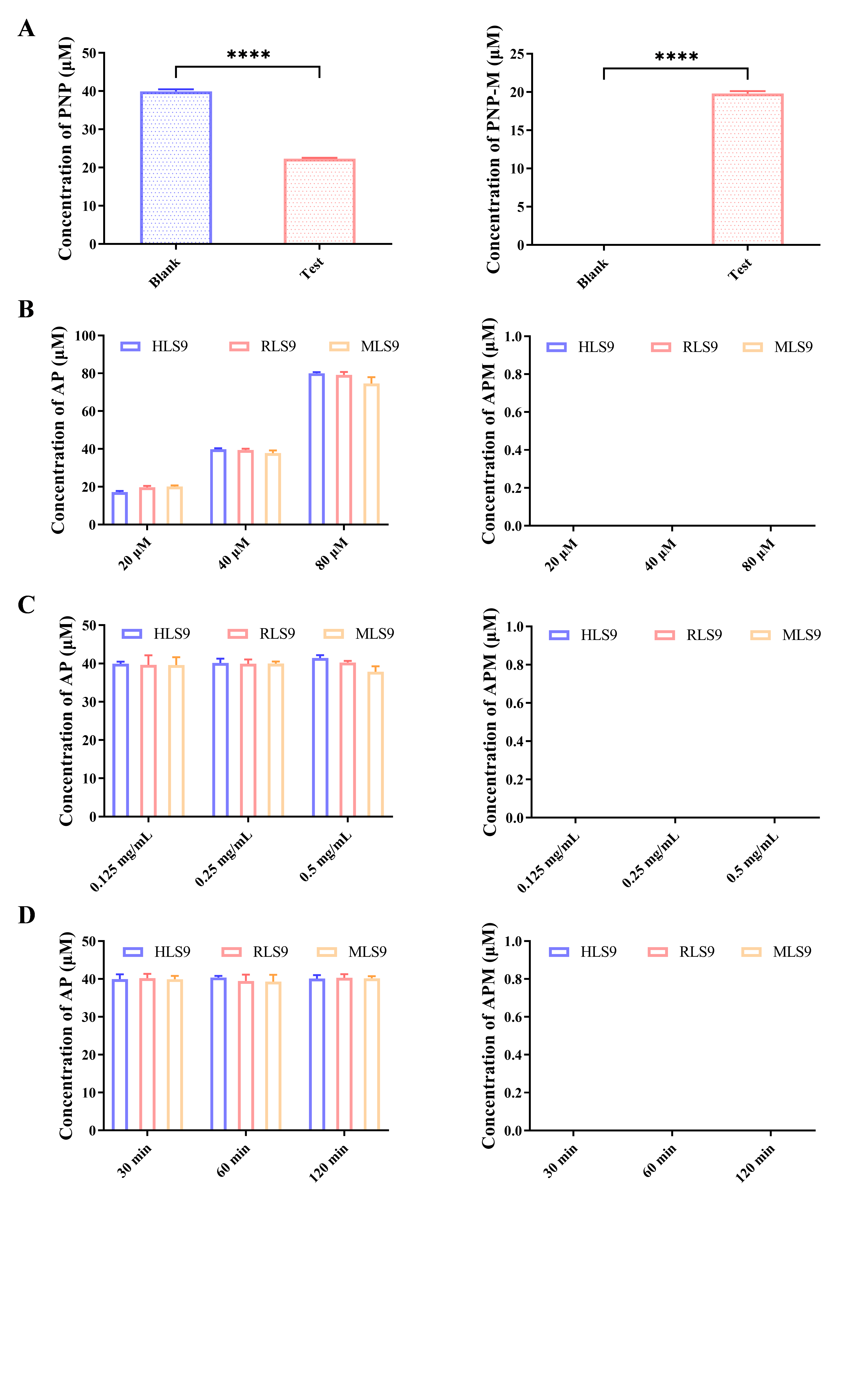


Supplementary Figure 2. Sulfonate metabolism of AP by human liver S9 fraction (HLS9), rat liver S9 fraction (RLS9), and mouse liver S9 fraction (MLS9). (A) sulfonate metabolism of P-nitrophenol (PNP, a classical substrate for SULTs) and its metabolite (PNP-M) by HLS9. (B-D) The impact of different AP concentrations (B), protein concentrations (C), and incubation time (D) on AP’s C-sulfonate metabolism. Data are presented as mean ± SEM (n = 3 per group). *****p* < 0.0001.


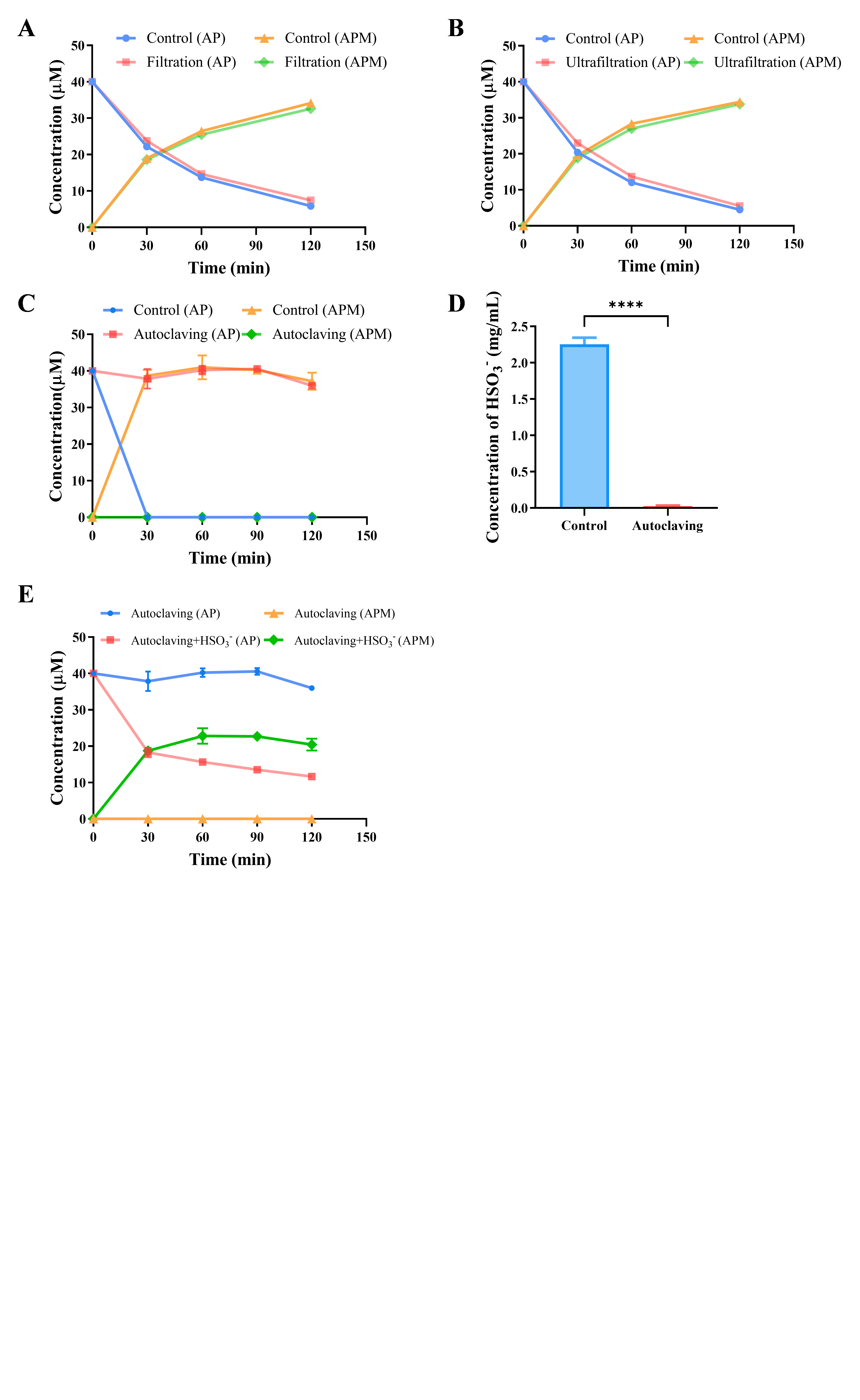


Supplementary Figure 3. The effect of HSO_3_^-^ on the C-sulfonate metabolism of AP in vitro. (A-B) Concentrations of AP and APM in filtration and ultrafiltration perfusate. (C) Concentrations of AP and APM in autoclaving perfusate. (D) Concentration of HSO_3_^-^ in autoclaving perfusate. (E) Concentrations of AP and APM in autoclaving perfusate supplemented with HSO_3_^-^. Data are presented as mean ± SEM (n = 3 per group). *****p* < 0.0001.


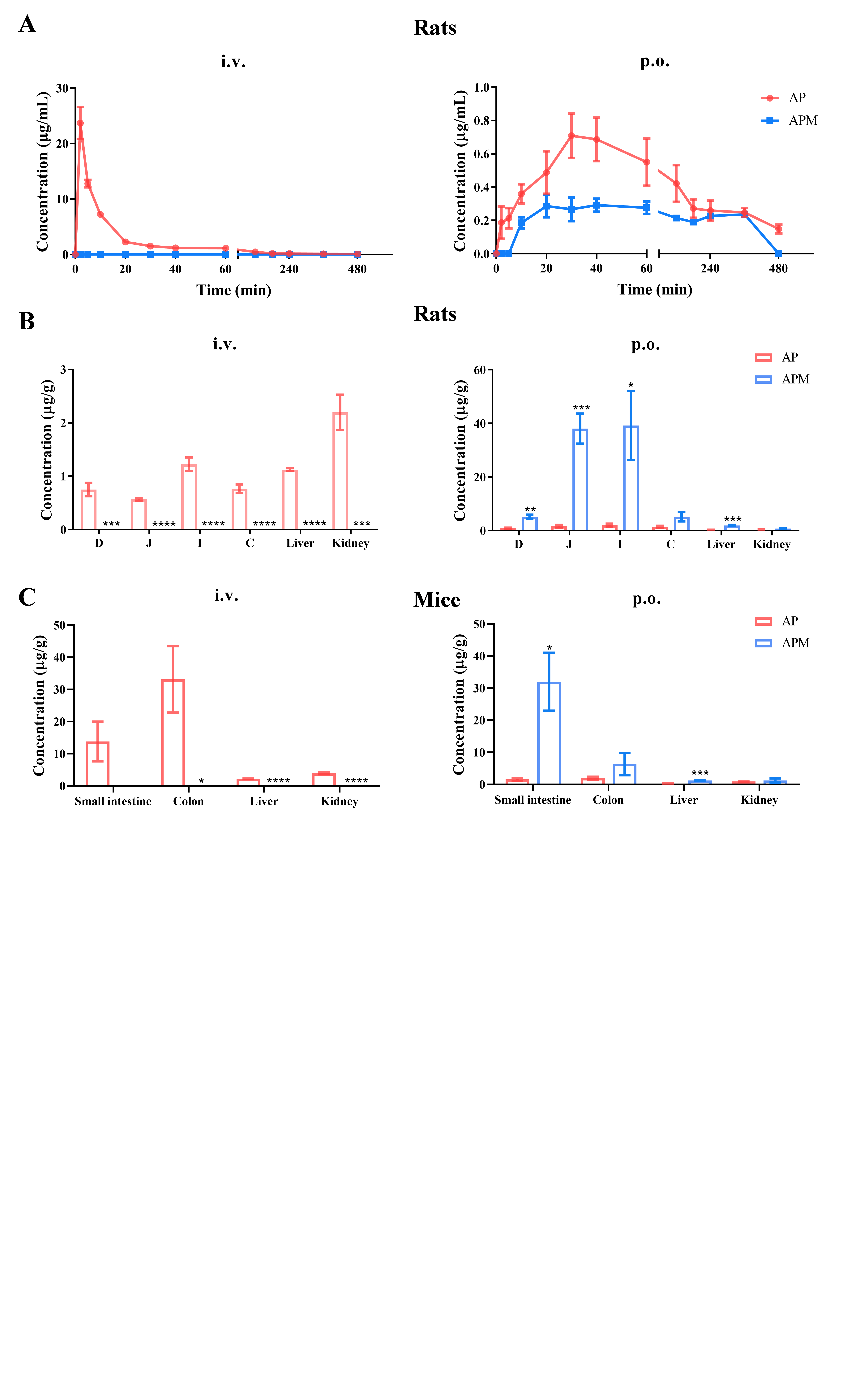


Supplementary Figure 4. C-sulfonate metabolism of AP after intravenous (i.v.) and oral (p.o.) administration of AP. (A) Plasma pharmacokinetics of AP and APM after intravenous administration of AP (24 mg/kg) and oral administration of AP (120 mg/kg) in rats (n = 5 per group). (B) Distribution of AP and APM in the duodenum (D), jejunum (J), ileum (I), colon (C), liver, and kidneys of rats after intravenous administration of AP (24 mg/kg) and oral administration of AP (120 mg/kg) (n = 4 per group). (C) Distribution of AP and APM in the small intestine, colon, liver, and kidneys of mice after intravenous administration of AP (20 mg/kg) and oral administration of AP (100 mg/kg) (n = 4 per group). Data are presented as mean ± SEM. **p* < 0.05, ***p* < 0.01, ****p* < 0.001 and *****p* < 0.0001.


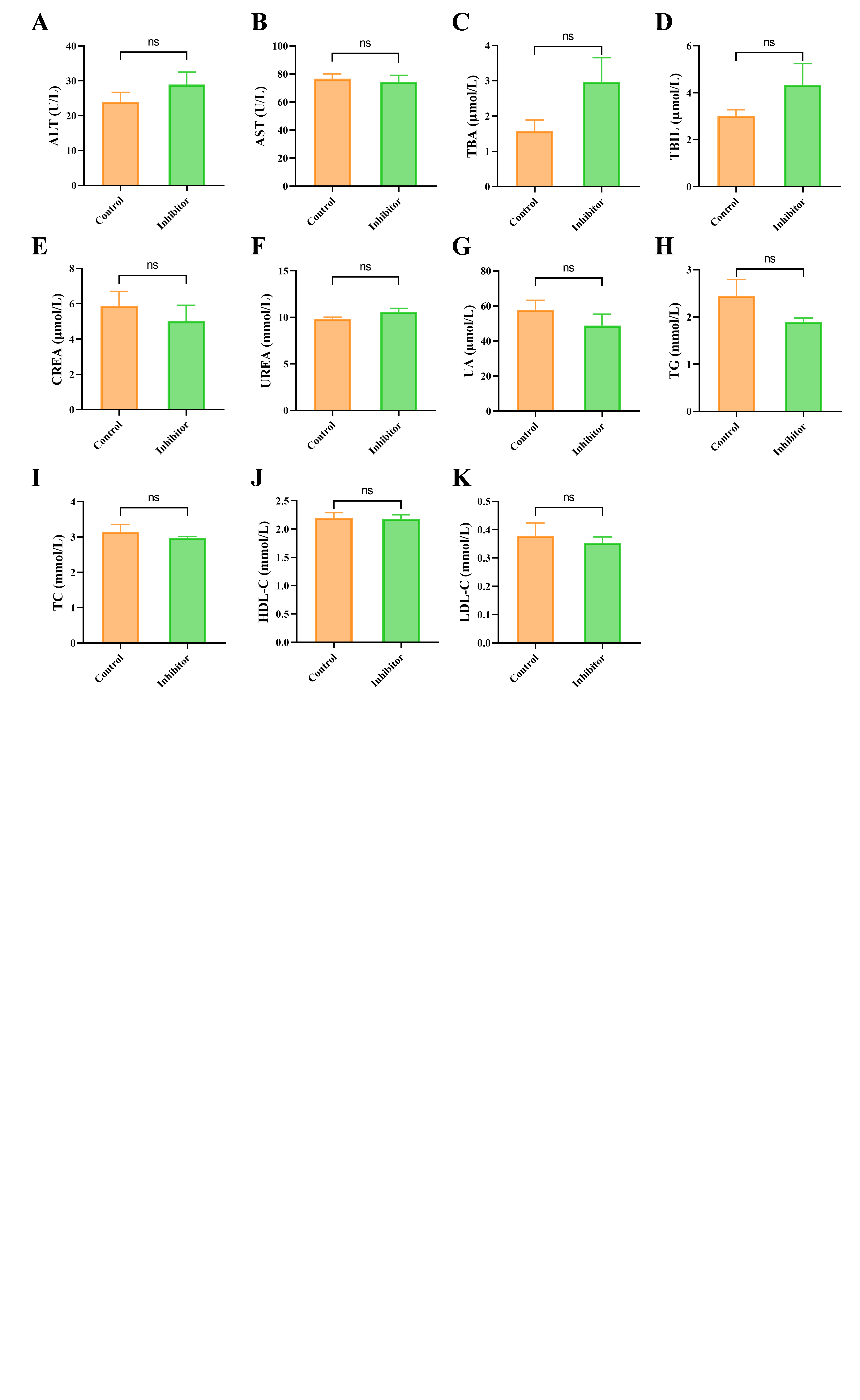


Supplementary Figure 5. The APS reductase inhibitor bromocriptine mesylate does not induce damage to other parts of the organism. (A-D) Liver function indicators including ALT, AST, TBA, and TBIL. (E-G) Renal function indicators including CREA, UREA, and UA. (H-K) Blood lipid indicators including TG, TC, HDL-C, and LDL-C. Inhibitor refers to the treatment with the APS reductase inhibitor bromocriptine mesylate at a dosage of 20 mg/kg, administered orally for 14 days in mice. Data are presented as mean ± SEM (n = 8 per group).


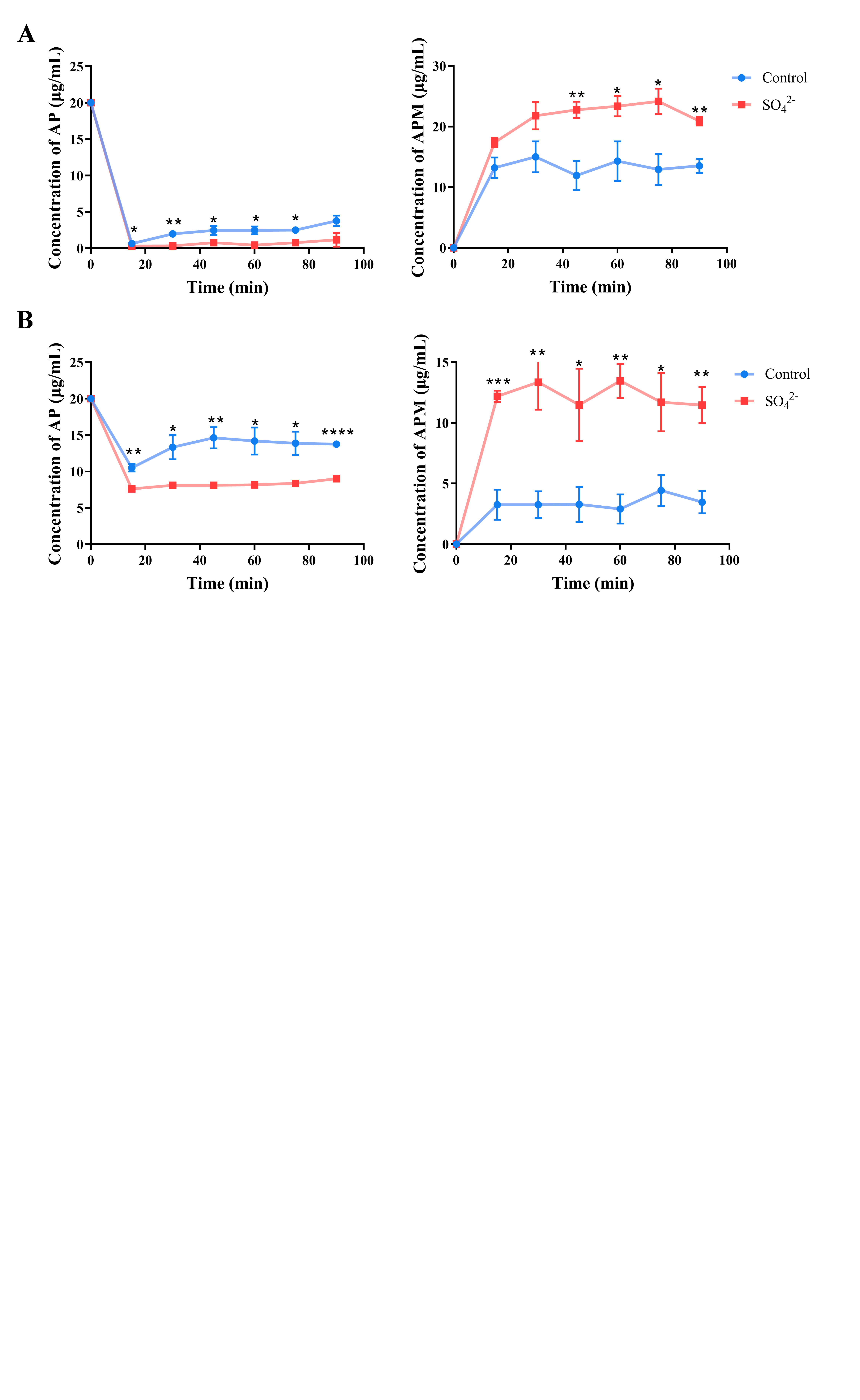


Supplementary Figure 6. The effect of SO_4_^2-^ on the C-sulfonate metabolism of AP through an in-situ intestinal perfusion experiment. (A) The concentrations of AP and APM in the duodenum during single-pass intestinal perfusion in rats (n = 4 per group). (B) The concentrations of AP and APM in the jejunum during single-pass intestinal perfusion in rats (n = 4 per group). SO_4_^2-^ refers to the co-perfusion of Na_2_SO_4_ with AP. Data are presented as mean ± SEM. **p* < 0.05, ***p* < 0.01, ****p* < 0.001 and *****p* < 0.0001.


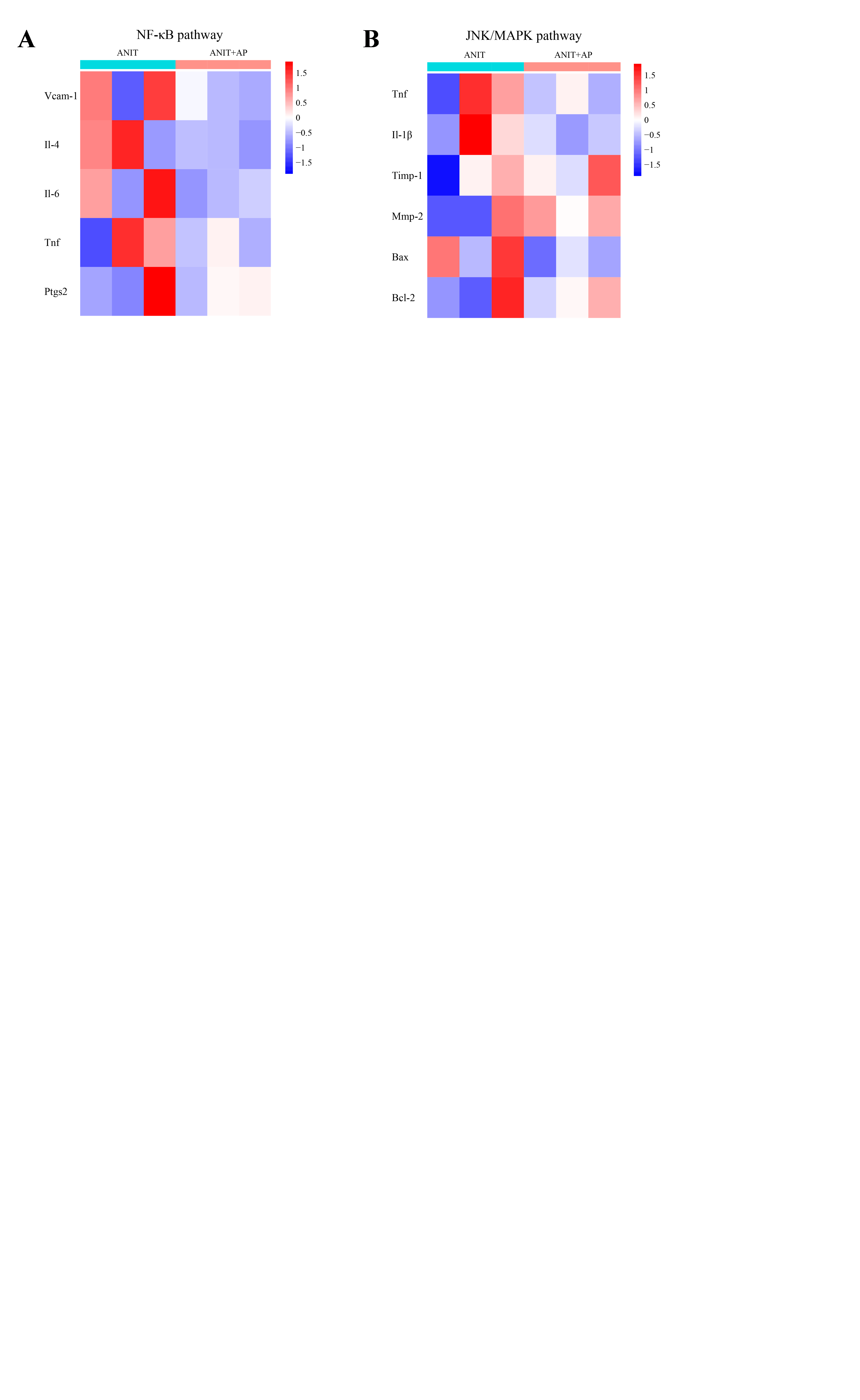


Supplementary Figure 7. Changes in NF-κB and JNK/MAPK pathways following AP treatment. (A-B) Heatmap for gene expression of NF-κB and JNK/MAPK pathways obtained from RNA sequencing analysis on liver samples collected from mice treated with ANIT and ANIT+AP (n = 3 per group).


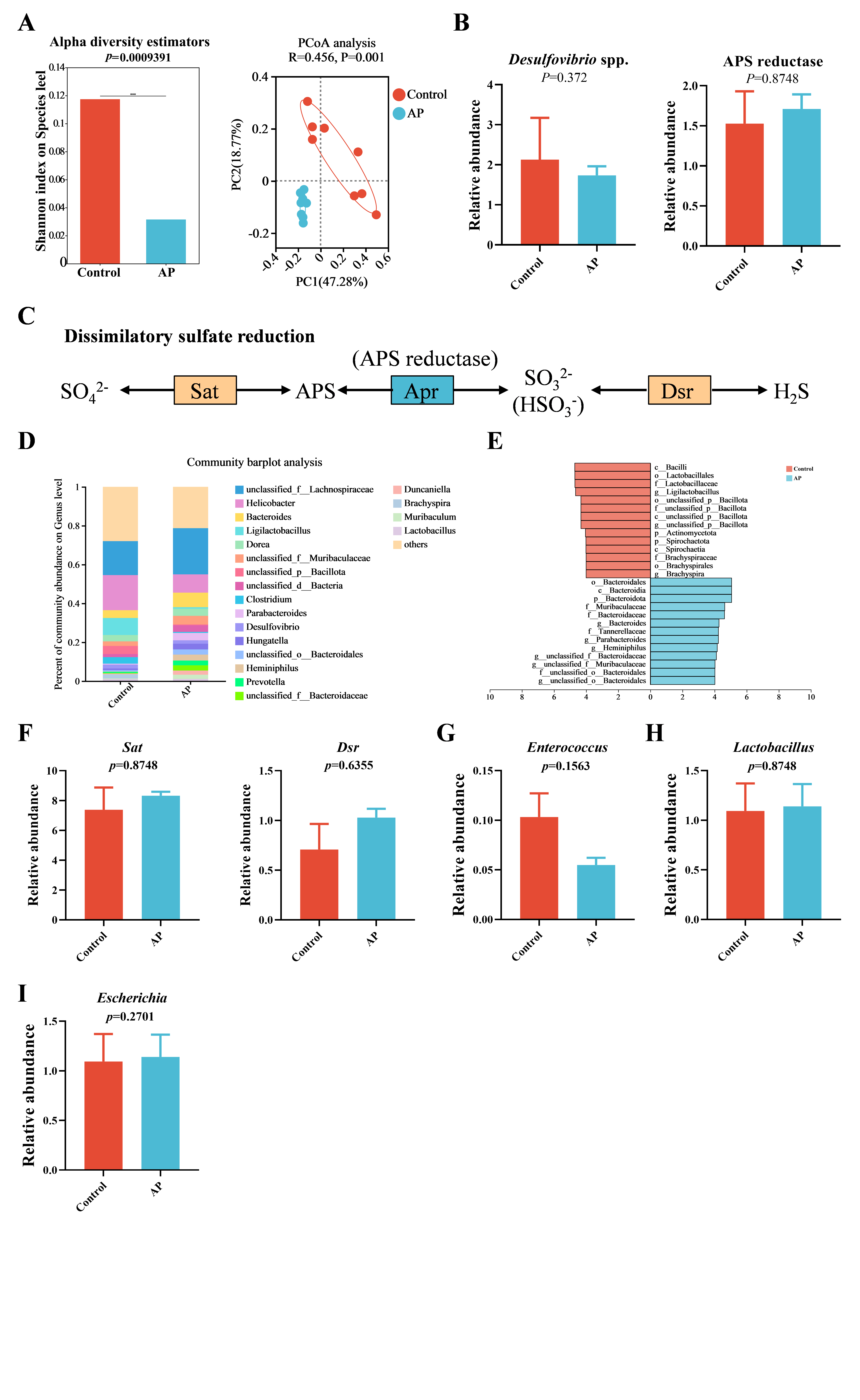


Supplementary Figure 8. The effect of AP on gut microbiota and dissimilatory sulfate reduction pathway. (A) α-diversity and β-diversity analysis using shotgun metagenomic sequencing on cecum samples following AP treatment (400 mg/kg, p.o., 14 days) mice (n = 8 per group). (B) Relative abundance of *Desulfovibrio* spp. and APS reductase in the cecum of mice after AP treatment by shotgun metagenomic sequencing (n = 8 per group). (C) Dissimilatory sulfate reduction pathway. (D) Percentage composition of the community on the genus level in the cecum of mice after AP (400 mg/kg, p.o., 14 days) treatment as determined by shotgun metagenomic sequencing (n = 8 per group). (E) Linear discriminant analysis effect size (LEfSe) analysis in the cecum of mice after AP treatment using shotgun metagenomic sequencing (n = 8 per group). (F) Relative abundance of gene coding for sulfate adenylyltransferase (*Sat*) (EC 2.7.7.4) and dissimilatory sulfite reductase alpha subunit (*Dsr*) (EC 1.8.1.22) involved in dissimilatory sulfate reduction route (n = 8 per group). (G-I) Relative abundance of *Enterococcus*, *Lactobacillus*, and *Escherichia* bacteria in the cecum of mice after AP treatment by shotgun metagenomic sequencing (n = 8 per group). Data are presented as mean ± SEM.

### **Methods**

### 1 Identification of the efficacy of antibiotic (ABX) in eliminating gut microbiota.

Cultivation and count of viable bacteria: To assess the efficacy of ABX in eliminating gut microbiota, fresh feces samples from ABX-treated and Control groups animals were collected in pre-weighed tubes containing 1 mL phosphate-buffered saline (PBS), followed by weighing. Subsequently, these samples were vortexed, filtered, and diluted with PBS to a 1:10 ratio. For cultivation and count of fecal bacteria, the diluted samples were further diluted to a 1:1000 ratio with PBS. Equal volumes (100 µL) of the diluted fecal suspensions from both ABX-treated and Control groups were then plated on Luria-Bertani (LB) agar plates and incubated for 48h^1^. The number of viable bacteria was counted using Image J software (V1.8.0, National Institutes of Health, USA).

### Quantification of total bacteria: DNA was extracted from a fixed amount of frozen feces (200 mg) using the TIANamp Stool DNA Kit (TIANGEN, Beijing, China) following the manufacturer’s protocol. PCR reaction mixtures were prepared as reported in the literature^2^. The PCR amplification of the 16S rRNA gene from total bacteria was conducted using the primer sets as listed in supplementary Table 1. The reaction included an initial denaturation at 95°C for 2 min, followed by cycles of denaturation at 95°C for 30 s, annealing at 60°C for 30 s, and elongation at 72°C for 1 min, with a final elongation at 72°C for 3 min. PCR product detection was performed using agarose gel electrophoresis and UV transillumination^3^.

### 2 AP incubation with liver S9 fraction

A sulfonate reaction system and incubation procedures were followed those of a previous study^4^. Briefly, the reaction mixture (total volume 200 μL) consists of 10 μL enzymes (MLS9, RLS9 and HLS9 at different concentrations), 5 μL magnesium chloride (1 mM), 5 μL 3’-phosphoadenosine-5’-phosphosulfate (PAPS, 0.05 mM), 178 μL potassium phosphate (KPI) buffer (44.5 mM, pH 7.4) and 2 μL AP at different concentrations. The mixture was prepared in triplicates and incubated in a shaking water bath at 37°C for various durations. The reactions were terminated by the addition of acetonitrile solution.

3 AP incubation with duodenal perfusate

To investigate whether HSO_3_^-^ is the essential ligand for the C-sulfonate metabolism of AP, duodenal perfusate of SD rat was collected according to the method described in the literature^5^. The duodenal perfusate was filtered by 0.22 μm Millex® needle filter, ultrafiltered by Amicon® Ultra filter or placed in an autoclave at 121°C for 2 hours, and then incubated with AP (40 μM) in vitro for 30, 60, 90 and 120 min. In another experiment, duodenal perfusate was autoclaved and then combined with HSO_3_^-^ (80 μM) and incubated with AP (40 μM) in vitro for 120 min. At the end of the incubation, 100 μL of ice-cold acetonitrile was quickly added to terminate the reaction. After centrifugation at 13000 rpm for 30 min, the supernatant was collected for UPLC-MS/MS analysis.

### 4 Pharmacokinetics study of AP by tail vein injection and oral administration

AP was administered intravenously and orally to detect the production of the C-sulfonate metabolite APM. In tissue distribution experiment, rats were given AP intravenously (24 mg/kg) or orally (120 mg/kg), while mice received AP intravenously (20 mg/kg) or orally (100 mg/kg). After a 90-minute interval, the animals were sacrificed, and their liver, kidneys, small intestine, and colon were collected for analysis. In pharmacokinetic studies, rats were administered 24 mg/kg of AP intravenously or 120 mg/kg of AP by oral gavage. Blood samples were collected at predetermined time intervals (0, 5, 10, 20, 30, 40, 60, 90, 120, 180, 240, 360, and 480 min), as described, to detect the content of AP and APM by UPLC-MS/MS.

### 5 The effect of SO_4_^2-^ on the C-sulfonate metabolism of AP through an in-situ intestinal perfusion experiment

The rat intestinal perfusion experiment was conducted based on previous literature reports^6, 7^. The experimental animals were divided into two groups: Control and SO_4_^2-^ groups. Rats were anaesthetised via the intraperitoneal injection of 50% urethane at a dose of 2.4 mL/kg. Two segments of the intestine, specifically the duodenum and jejunum, each measuring 5-10 cm in length, were cannulated simultaneously. The primary tube was affixed to a syringe controlled by an infusion pump (Model PHD2000 Harvard Apparatus, Cambridge, Massachusetts). The inlet cannula was insulated and maintained in a constant temperature of 37°C in circulating water bath to ensure the perfusate’s thermal stability. Following the surgical procedure, the two segments of Control group were perfused with 20 μM AP, and the two segments of SO_4_^2-^ group were co-perfused with AP (20 μM) and Na_2_SO_4_ (40 μM). The perfusion was conducted at the flow rate of 0.17 mL/min and perfusate samples were collected every 15 min (15, 30, 45, 60, 75, and 90 min). The concentrations of AP and APM in the perfusate at the outlet were determined through UPLC-MS/MS analysis.

Supplementary Table 1. The primer sequences used for RT-qPCR assay in mice.

| Primers | Forward 5’-3’ | Reverse 5’-3’ |
| --- | --- | --- |
| *β-actin* | GACGGCCAGGTCATCACTATTG | AGGAAGGCTGGAAAAGAGCC |
| *Fxr* | GGCCTCTGGGTACCACTACA | TGTACACGGCGTTCTTGGTA |
| *Mrp2* | GCACTGTAGGCTCTGGGAAG | TGCTGAGGGACGTAGGCTAT |
| *Mrp3* | AGAGCTGGGCTCCAAGTTCT | TGGTGTCTCAGGTAAAACAGGTAGCA |
| *Mrp4* | CATCAAGTCCAGGGAAAAGGTTG | GAGGGCCGAGATGAGGGAG |
| *Ost-α* | ACGGTGGTGTCTGTGTTCTG | GGTGTCCTTCAGTGTCCTTAGTA |
| *Ost-β* | GTATTTTCGTGCAGAAGATGCG | TTTCTGTTTGCCAGGATGCTC |
| *Ntcp* | CACCATGGAGTTCAGCAAGA | CCAGAAGGAAAGCACTGAGG |
| *Oatp1* | ATCCAGTGTGTGGGGACAAT | GCAGCTGCAATTTTGAAACA |
| *Bsep* | CTGCCAAGGATGCTAATGCA | CGATGGCTACCCTTTGCTTCT |
| Total bacteria | ACTCCTACGGGAGGCAGCAG | ATTACCGCGGCTGCTGG |
| APS reductase | TGGCAGATCATCATCAACGG | TTGAACAGCAGGAACCARCCRCC |
| *Desulfovibrio* spp. | CCGTAGATATCTGGAGGAACATCAG | ACATCTAGCATCCATCGTTTACAGC |
| *D. piger* | CTAGGGTGTTCTAATCATCATCCTAC | GATATCTACGGATTTCACTCCTACACC |

**References**

1. Zarrinpar A, Chaix A, Xu ZZ, Chang MW, Marotz CA, Saghatelian A, et al. Antibiotic-induced microbiome depletion alters metabolic homeostasis by affecting gut signaling and colonic metabolism. Nat Commun. 2018; 9(1):2872. <http://dx.doi.org/10.1038/s41467-018-05336-9>

2. Kuno T, Hirayama-Kurogi M, Ito S, Ohtsuki S. Reduction in hepatic secondary bile acids caused by short-term antibiotic-induced dysbiosis decreases mouse serum glucose and triglyceride levels. Sci Rep. 2018; 8(1):1253. <http://dx.doi.org/10.1038/s41598-018-19545-1>

3. Vitali B, Biagi E, Brigidi P. Protocol for the use of PCR-denaturing gradient gel electrophoresis and quantitative PCR to determine vaginal microflora constitution and pathogens in bacterial vaginosis. Methods Mol Biol. 2012; 903:177-193. <http://dx.doi.org/10.1007/978-1-61779-937-2_11>

4. Zhang Q, Zhu L, Gong X, Ruan Y, Yu J, Jiang H, et al. Sulfonation Disposition of Acacetin: In Vitro and in Vivo. J Agric Food Chem. 2017; 65(24):4921-4931. <http://dx.doi.org/10.1021/acs.jafc.7b00854>

5. Dahlgren D, Roos C, Peters K, Lundqvist A, Tannergren C, Sjögren E, et al. Evaluation of drug permeability calculation based on luminal disappearance and plasma appearance in the rat single-pass intestinal perfusion model. Eur J Pharm Biopharm. 2019; 142:31-37. <http://dx.doi.org/10.1016/j.ejpb.2019.06.011>

6. Ye L, Lu L, Li Y, Zeng S, Yang X, Chen W, et al. Potential role of ATP-binding cassette transporters in the intestinal transport of rhein. Food Chem Toxicol. 2013; 58:301-305. <http://dx.doi.org/10.1016/j.fct.2013.04.044>

7. Ye L, Wang T, Tang L, Liu W, Yang Z, Zhou J, et al. Poor oral bioavailability of a promising anticancer agent andrographolide is due to extensive metabolism and efflux by P-glycoprotein. J Pharm Sci. 2011; 100(11):5007-5017. <http://dx.doi.org/10.1002/jps.22693>
