# Supplementary figures and images for: Gut microbiota-mediated C-sulfonate metabolism impairs the bioavailability and anti-cholestatic efficacy of andrographolide
Source: Gut Microbes. 2024 Sep 12;16(1):2387402. doi: 10.1080/19490976.2024.2387402 (PMC11404609; doi:10.1080/19490976.2024.2387402)

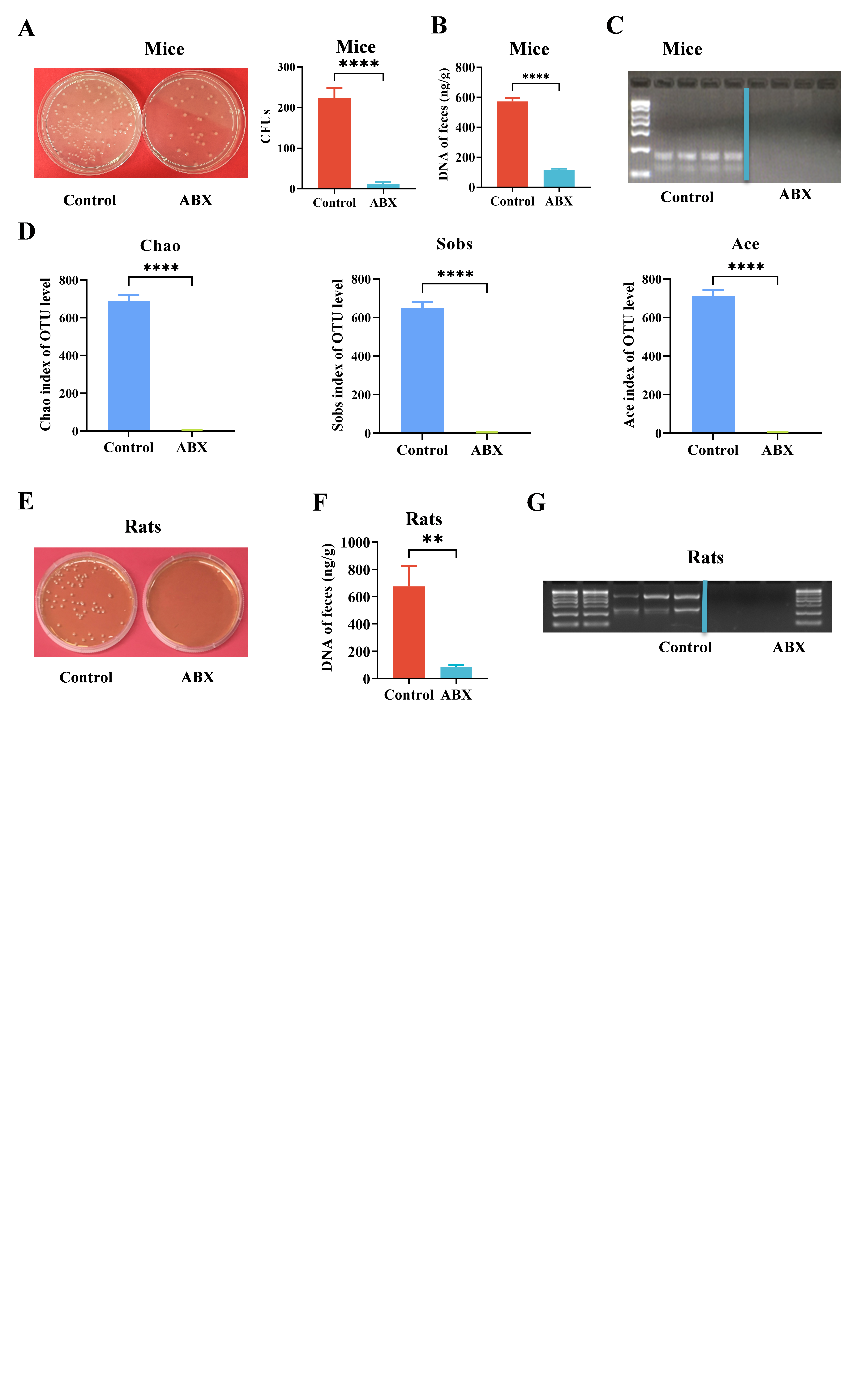

Supplement: Supplemental Material [file KGMI_A_2387402_SM4329.zip › Supplementary Figure 1 (2).tif]

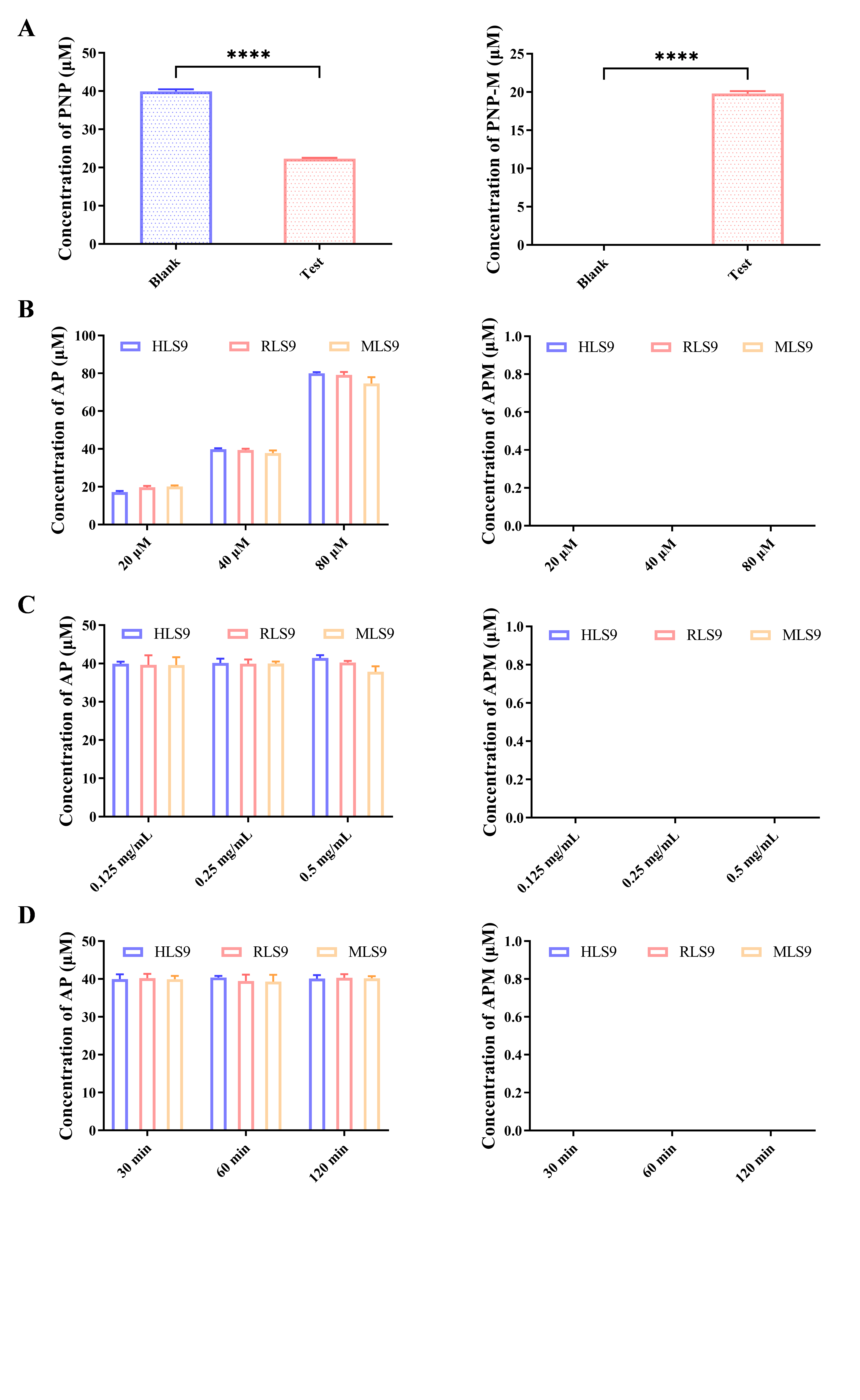

Supplement: Supplemental Material [file KGMI_A_2387402_SM4329.zip › Supplementary Figure 2 (1).tif]

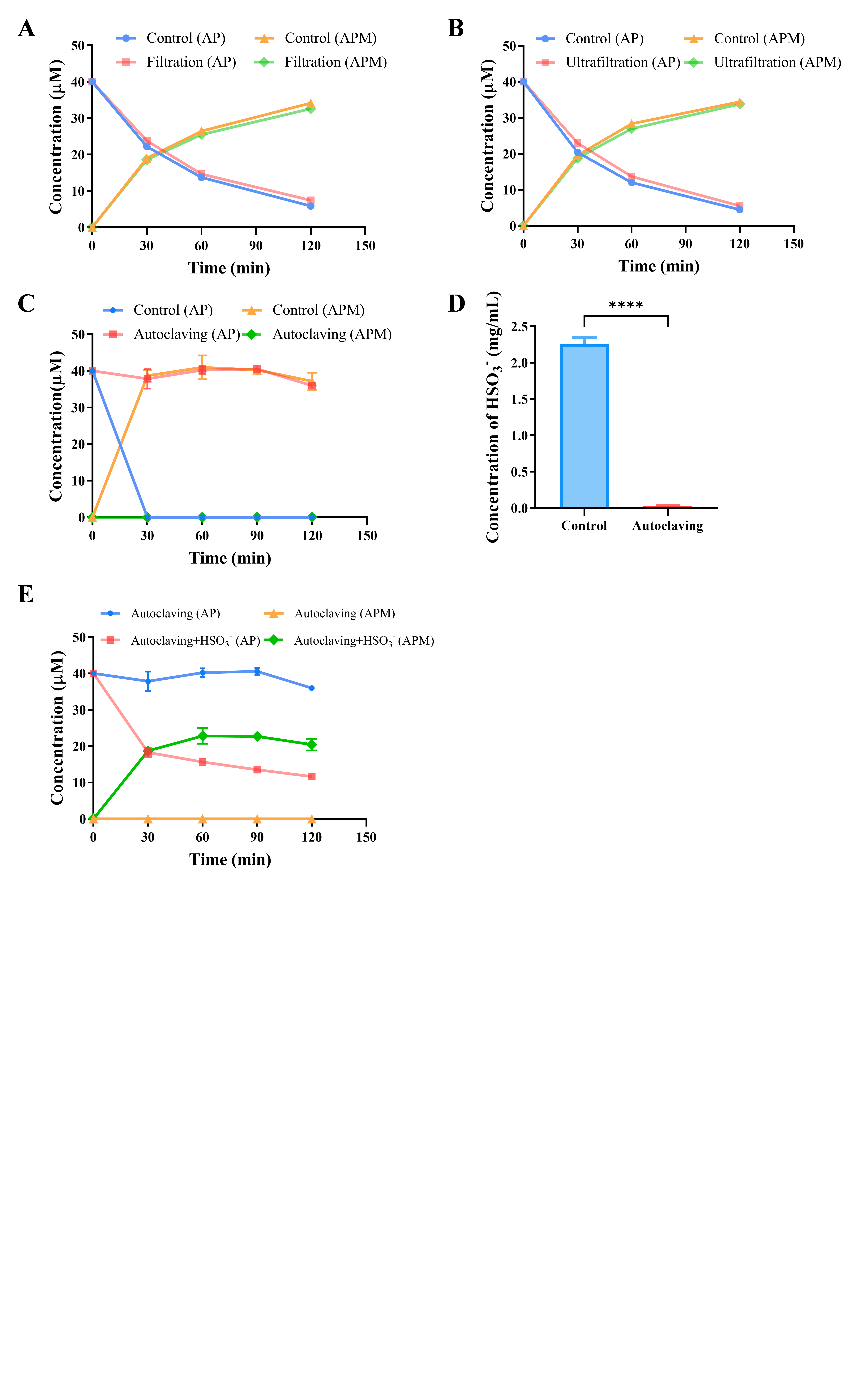

Supplement: Supplemental Material [file KGMI_A_2387402_SM4329.zip › Supplementary Figure 3 (1).tif]

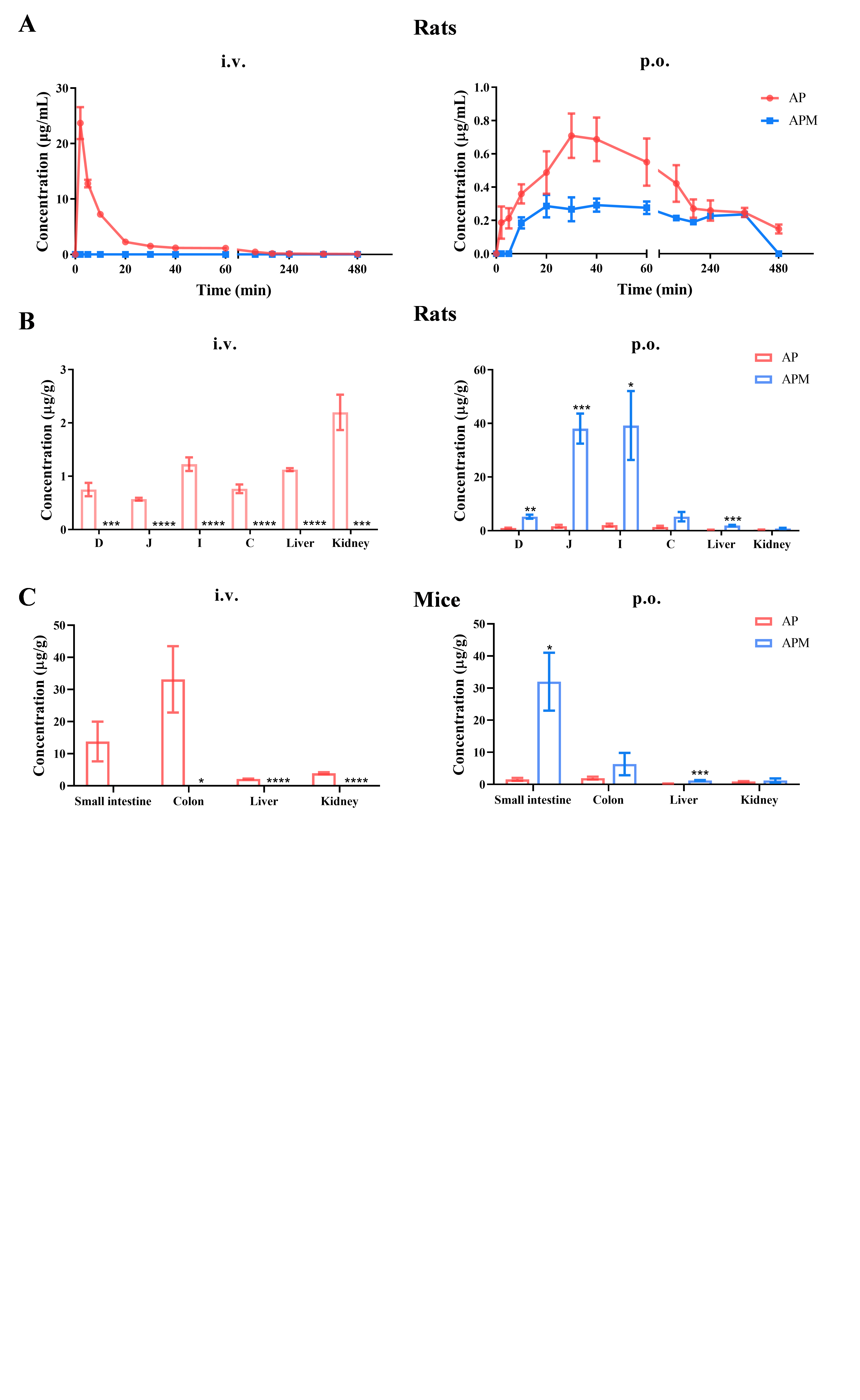

Supplement: Supplemental Material [file KGMI_A_2387402_SM4329.zip › Supplementary Figure 4 (1).tif]

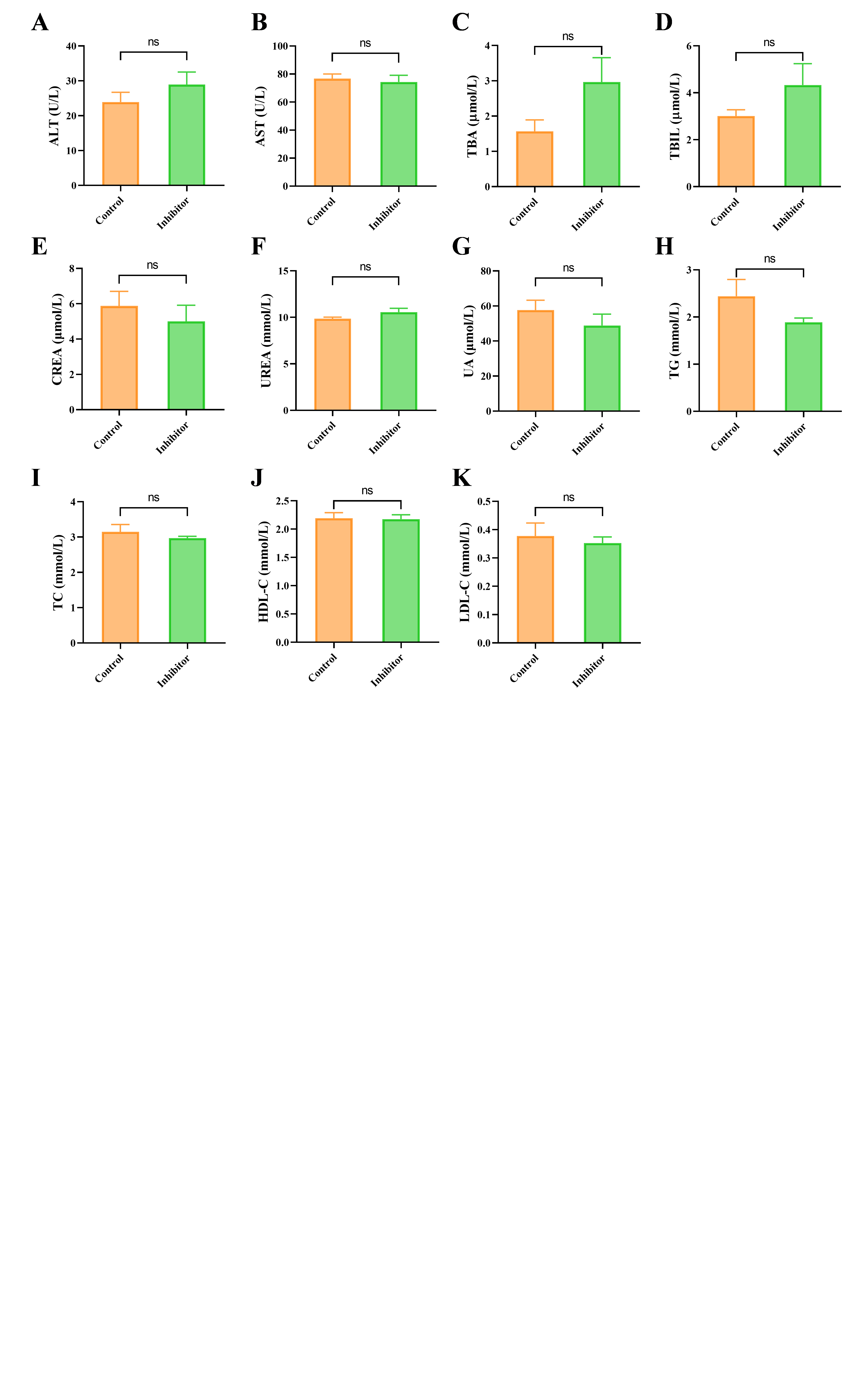

Supplement: Supplemental Material [file KGMI_A_2387402_SM4329.zip › Supplementary Figure 5 (1).tif]

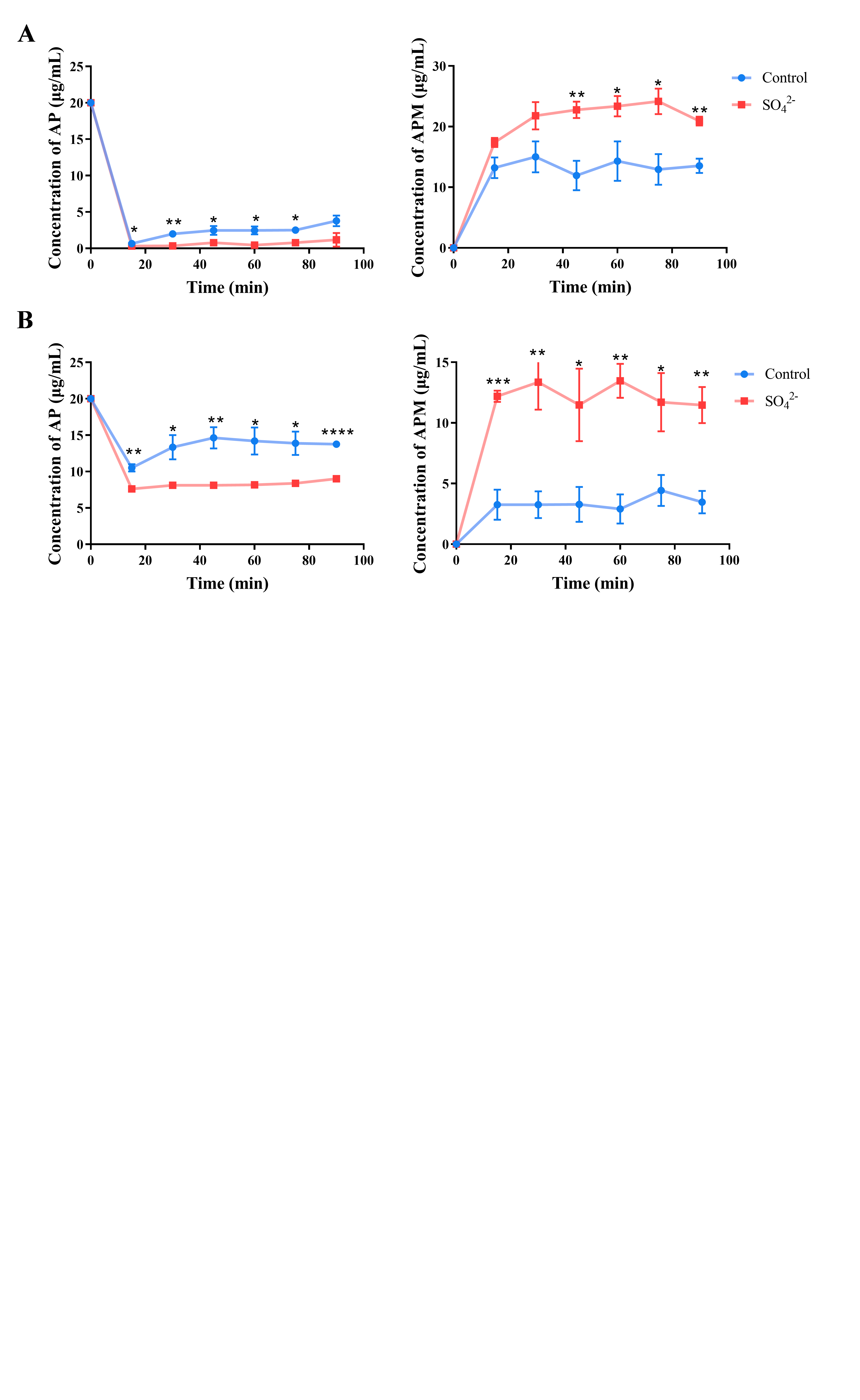

Supplement: Supplemental Material [file KGMI_A_2387402_SM4329.zip › Supplementary Figure 6 (1).tif]

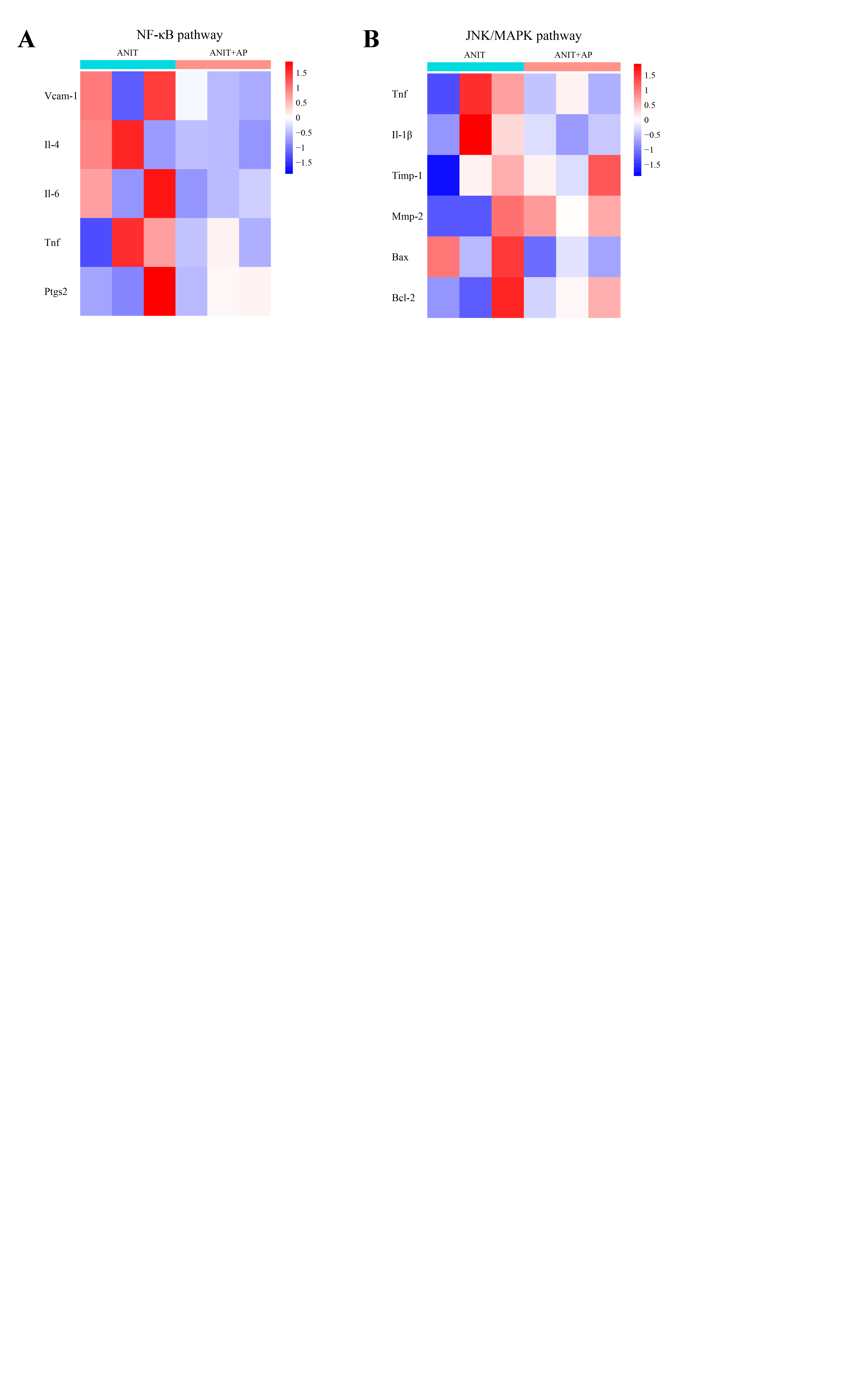

Supplement: Supplemental Material [file KGMI_A_2387402_SM4329.zip › Supplementary Figure 7 (1).tif]

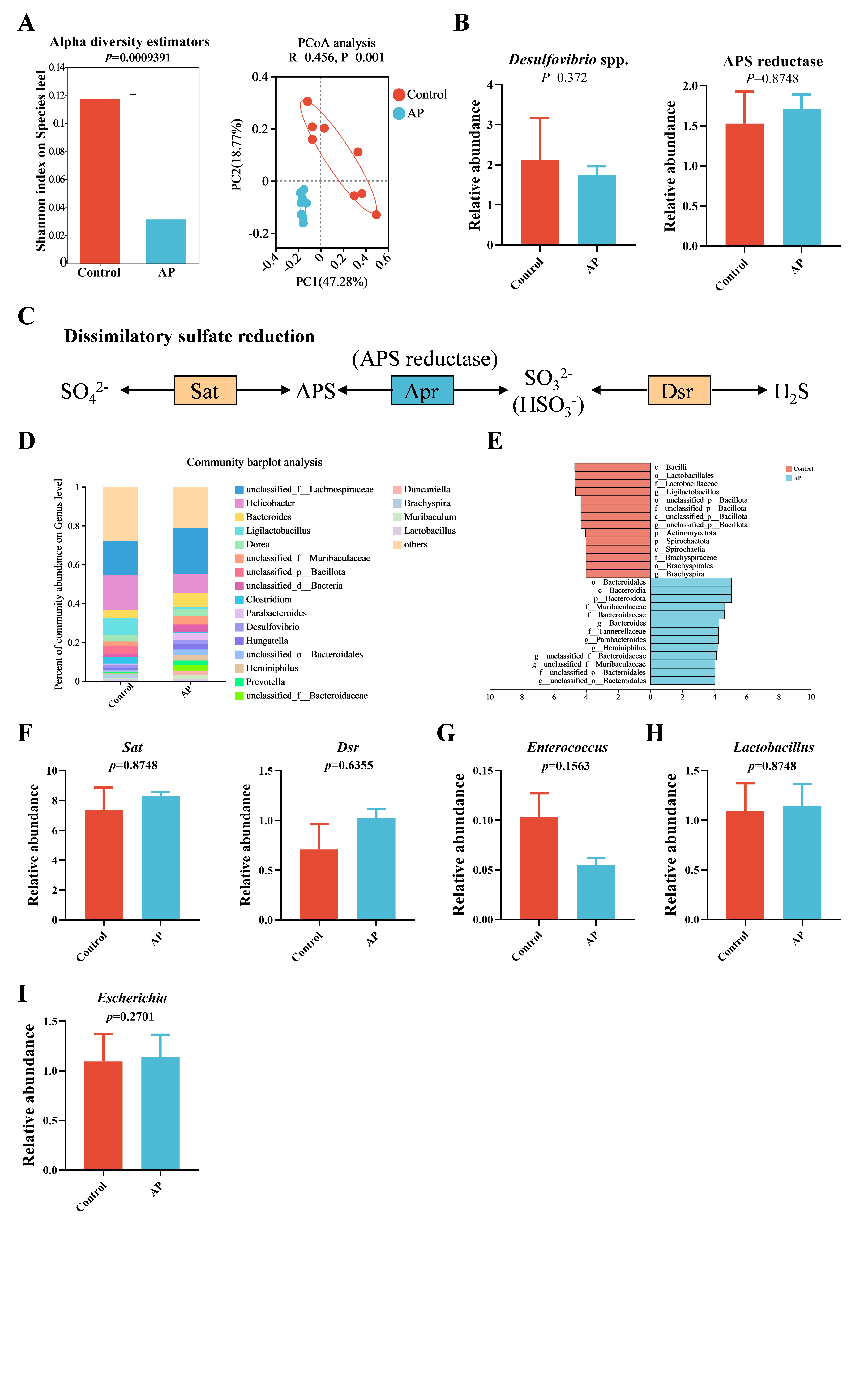

Supplement: Supplemental Material [file KGMI_A_2387402_SM4329.zip › Supplementary Figure 8 (1).tif]
